# Supplementary material for: Quantum variational algorithms are swamped with traps
Source: Nat Commun. 2022 Dec 15;13:7760. doi: 10.1038/s41467-022-35364-5 (PMC9755303; doi:10.1038/s41467-022-35364-5)
Supplement: Supplementary file 1 — Supplementary Information [file 41467_2022_35364_MOESM1_ESM.pdf]

Supplementary Information

Eric R. Anschuetz*[1] and Bobak T. Kiani†[2]

[1]MIT Center for Theoretical Physics, 77 Massachusetts Avenue, Cambridge, MA 02139, USA

[2]MIT Department of Electrical Engineering and Computer Science, 77 Massachusetts Avenue, Cambridge, MA 02139, USA

Supplementary Note 1– Training Error Dominates in the Optimization of Variational Quantum Algorithms

The variational quantum eigensolver [1] is purely a problem of optimization and may appear unrelated to the challenges in learning via variational algorithms; however, by decomposing the error of a learning algorithm into key terms using well-established methods [2], we will show that variational learning algorithms essentially face the same optimization task and its associated challenges. In both cases, the hardness of learning or optimizing with variational circuits manifests itself in the challenges of optimizing over a cost landscape riddled with traps (or other barriers to optimization).

We restrict ourselves here to the supervised learning framework of empirical risk minimization, where our goal is to learn a space of input and output pairs $(\mathbf{x}, \mathbf{y}) \in \mathcal{X} \times \mathcal{Y}$ drawn from a distribution $P(\mathbf{x}, \mathbf{y})$. Given a loss function $\ell : \mathcal{Y} \times \mathcal{Y} \to [0, \infty)$, we quantify how well our function performs by considering the expected risk $\mathcal{R}$:

$$\mathcal{R}(f) = \mathbb{E}_{\mathbf{x}} \left[\, \ell(f(\mathbf{x}), f^*(\mathbf{x})) \, \right], \tag{1}$$

where the expectation above is taken with respect to $P(\mathbf{x}, \mathbf{y})$. To benchmark performance, we compare to the "optimal" or target function f^* which is the minimizer of the risk:

$$f^*(\mathbf{x}) = \operatorname*{arg\,min}_{\hat{\mathbf{y}} \in \mathcal{Y}} \mathbb{E} \left[\ell(\mathbf{y}, \hat{\mathbf{y}}) | \mathbf{x} \right]. \tag{2}$$

To perform learning, we search for a function $\widehat{f} \in \mathcal{F}$ in the function class $\mathcal{F}$ (think *e.g.,* the set of functions expressed by quantum neural networks). The expected risk $\mathcal{R}(f)$ is not something one can calculate since it requires access to the full probability distribution of the data. Instead, one minimizes the empirical risk $\widehat{\mathcal{R}}(f)$ (often named the training error) over a given training data set $\mathcal{D}$ of size N consisting of pairs $\{\mathbf{x}_i, \mathbf{y}_i\}_{i=1}^N$:

$$\widehat{\mathcal{R}}(f) = \sum_{i=1}^{N} \ell(f(\mathbf{x}_i), f^*(\mathbf{x}_i)). \tag{3}$$

Note that we use the hat in $\widehat{\mathcal{R}}$ and $\widehat{f}$ to denote the expected risk measure and function that one actually has access to during training or optimization. Given the above, one can bound the expected risk of any

*eans@mit.edu

†bkiani@mit.edu

function $\widehat{f}$ as a decomposition below [2]:

$$\mathbb{E}\left[\mathcal{R}(\widehat{f})-\mathcal{R}(f^*)\right] \leq \underbrace{\min_{f\in\mathcal{F}}\mathcal{R}(f)-\mathcal{R}(f^*)}_{\text{approximation error}} + \underbrace{2\mathbb{E}\left[\sup_{f\in\mathcal{F}}\left|\mathcal{R}(f)-\widehat{\mathcal{R}}(f)\right|\right]}_{\text{generalization error}} + \underbrace{\mathbb{E}\left[\widehat{\mathcal{R}}(\widehat{f})-\min_{f\in\mathcal{F}}\widehat{\mathcal{R}}(f)\right]}_{\text{optimization error}}, \tag{4}$$

where the expectation above is taken with respect to the distribution over data sets or training sets. The proof of this statement follows by a careful, yet straightforward, application of additions/subtractions with corresponding bounds [2].

Proof. Let $\widehat{f}_{\mathcal{F}} = \arg\min_{f\in\mathcal{F}}\widehat{\mathcal{R}}(f)$ and $f_{\mathcal{F}} = \arg\min_{f\in\mathcal{F}}\mathcal{R}(f)$. Then, by adding and subtracting quantities, we obtain the following result:

$$\begin{aligned}\mathbb{E}\left[\mathcal{R}(\widehat{f})-\mathcal{R}(f^*)\right] = \mathbb{E}\big[&\mathcal{R}(\widehat{f})-\mathcal{R}(f^*)\\ &+\widehat{\mathcal{R}}(\widehat{f}_{\mathcal{F}})-\widehat{\mathcal{R}}(\widehat{f}_{\mathcal{F}})\\ &+\mathcal{R}(f_{\mathcal{F}})-\mathcal{R}(f_{\mathcal{F}})+\widehat{\mathcal{R}}(f_{\mathcal{F}})-\widehat{\mathcal{R}}(f_{\mathcal{F}})\\ &+\widehat{\mathcal{R}}(\widehat{f})-\widehat{\mathcal{R}}(\widehat{f})\big].\end{aligned} \tag{5}$$

We reorder the above as follows and note their relation to the main statement:

$$\begin{aligned}\mathbb{E}\left[\mathcal{R}(\widehat{f})-\mathcal{R}(f^*)\right] = \ &\mathbb{E}\left[\mathcal{R}(f_{\mathcal{F}})-\mathcal{R}(f^*)\right] && \text{approximation error} \quad (6)\\ &+\mathbb{E}\left[\mathcal{R}(\widehat{f})-\widehat{\mathcal{R}}(\widehat{f})\right] && \text{generalization error} \quad (7)\\ &+\mathbb{E}\left[\widehat{\mathcal{R}}(f_{\mathcal{F}})-\mathcal{R}(f_{\mathcal{F}})\right] && \text{generalization error} \quad (8)\\ &+\mathbb{E}\left[\widehat{\mathcal{R}}(\widehat{f}_{\mathcal{F}})-\widehat{\mathcal{R}}(f_{\mathcal{F}})\right] && \leq 0 \text{ since } \widehat{f}_{\mathcal{F}} \text{ minimizes } \widehat{\mathcal{R}} \quad (9)\\ &+\mathbb{E}\left[\widehat{\mathcal{R}}(\widehat{f})-\widehat{\mathcal{R}}(\widehat{f}_{\mathcal{F}})\right] && \text{optimization error} \quad (10)\end{aligned}$$

For the quantities in the generalization error, we have since $\widehat{f}, f_{\mathcal{F}} \in \mathcal{F}$:

$$\begin{aligned}\mathbb{E}\left[\mathcal{R}(\widehat{f})-\widehat{\mathcal{R}}(\widehat{f})\right] &\leq \mathbb{E}\left[\sup_{f\in\mathcal{F}}\left|\mathcal{R}(f)-\widehat{\mathcal{R}}(f)\right|\right]\\ \mathbb{E}\left[\widehat{\mathcal{R}}(f_{\mathcal{F}})-\mathcal{R}(f_{\mathcal{F}})\right] &\leq \mathbb{E}\left[\sup_{f\in\mathcal{F}}\left|\mathcal{R}(f)-\widehat{\mathcal{R}}(f)\right|\right].\end{aligned} \tag{11}$$

Plugging these into equation 5 and noting as before that $\mathbb{E}\left[\widehat{\mathcal{R}}(\widehat{f}_{\mathcal{F}})-\widehat{\mathcal{R}}(f_{\mathcal{F}})\right] \leq 0$, we arrive at the desired result.

In the context of quantum variational algorithms, each of these has the following properties:

- The **approximation error** quantifies how well the most optimal function in the hypothesis class $\mathcal{F}$ can fit the function. In variational settings, the approximation error is typically bounded by assuming the target function is generated from a nice class of functions (e.g. shallow circuits) or arguing either analytically or theoretically that a given ansatz can (approximately) express the target function [3, 4, 5, 6].

- The **generalization error** quantifies the statistical error that arises from having a finite data set and is typically insignificant in quantum variational algorithms where circuit complexity is limited with regards to the number of training samples. More precisely, for data sets of size m, previous work [7, 8] bound the generalization error as $\tilde{O}(\sqrt{|G|/m})$ where $|G|$ is the number of trainable gates. In contrast, generalization error in heavily overparameterized classical neural network models are challenging to bound and it is still an open question why deep learning models generalize so well [9, 10].

- The **optimization error** measures how well one is able to reduce the empirical risk. Issues with optimization such as poor local minima and barren plateaus arise here. Note that there is a distinct difference between quantum and classical deep learning here. With classical deep neural networks, this quantity is typically negligible since neural networks are overparameterized with respect to the data set size and can fit random data arbitrarily well [9, 11]. Furthermore, due to efficient means of calculating gradients with bit-level precision, classical machine learning algorithms perform optimization over parameters far more efficiently than quantum variational algorithms. In quantum variational models, overparameterization with respect to the Hilbert space dimension is generally needed to arbitrarily fit data [12, 13, 14]. Since the Hilbert space dimension grows exponentially with the number of qubits, such overparameterization becomes prohibitive rather rapidly.

In summary, the approximation error and generalization error can be bounded efficiently with sufficient data so failures in learning are typically related to optimization over the empirical risk. As an aside, this is loosely analogous to the classical setting of learning polynomial size Boolean circuits which is strongly conjectured to be hard since the space of Boolean functions is challenging to search over [15].

Finally, we would like to stress that the decomposition of the excess risk performed in this section is neither unique nor necessarily tight. The decomposition can be performed in various other ways depending on the quantities one would like to bound. We chose the decomposition here to relate errors in quantum machine learning algorithms to their classical counterparts and to highlight the challenges one may face when attempting to provably learn a target function class.

Supplementary Note 2– Statistical Query Framework: Background and Additional Details

The statistical query (SQ) framework was introduced nearly 25 years ago to analyze the hardness of learning problems [16]. This framework restricts algorithms to a series of noisy queries, and hardness results are stated in terms of the number of queries needed to learn a given class of functions. Since there are various different ways of defining the statistical query model—including a recent quantum oracular version proposed in [17]—let us first review some of the various models considered in prior work.

1. **Classical statistical query model**: Introduced by [16], this was the first statistical query model introduced. For a given distribution D of inputs over an input space X and target concept $c : X \to \{-1, +1\}$, one can make a statistical query $\mathrm{SQ}(q, \tau)$, by providing a threshold $\tau \in \mathbb{R}^+$ and a query function $q : X \times \{-1, +1\} \to \{-1, +1\}$. The query returns a value in the range:

$$\mathbb{E}_{x\sim D}\left[q(x, c(x)) - \tau\right] \leq \mathrm{SQ}(q, \tau) \leq \mathbb{E}_{x\sim D}\left[q(x, c(x)) + \tau\right]. \tag{12}$$

2. **Correlational statistical query model**: The query is the same as before, except now, one queries correlations $\mathrm{CSQ}(q, \tau)$ only by providing a threshold $\tau \in \mathbb{R}^+$ and a query function $h : X \to \{-1, +1\}$. The query returns a value in the range:

$$\mathbb{E}_{x\sim D}\left[h(x)c(x) - \tau\right] \leq \mathrm{CSQ}(h, \tau) \leq \mathbb{E}_{x\sim D}\left[h(x)c(x) + \tau\right]. \tag{13}$$

   This model is strictly less powerful than the standard statistical query model since one can perform a correlational statistical query with a standard statistical query [18].

3. **Quantum statistical query model**: This is a statistical query model with quantum samples [17]. Here, we are restricted to target (classical) Boolean functions $c : \{0,1\}^n \to \{-1,+1\}$. A quantum statistical query $\text{Qstat}(\tau, M)$ is provided with a threshold $\tau \in \mathbb{R}^+$ and an observable or Hamiltonian $M \in (\mathbb{C}^2)^{n+1} \times (\mathbb{C}^2)^{n+1}$ satisfying $\|M\| \leq 1$ and returns a number in the range:

$$\langle\psi_c| M |\psi_c\rangle - \tau \leq \text{Qstat}(M, \tau) \leq \langle\psi_c| M |\psi_c\rangle + \tau, \tag{14}$$

where $|\psi_c\rangle = \sum_{x\in\{0,1\}^n} \sqrt{D(x)}\, |x\rangle\, |c(x)\rangle$. This model is useful to analyze the hardness of learning classical Boolean functions when given the extra power of querying the classical function in superposition. Our work considers learning quantum data and thus does not fit into the framework of this SQ model.

The SQ learning setting is related to the probably approximately correct (PAC) setting of learning theory [19] in that if an algorithm can learn a given function class in the SQ learning setting under any input distribution, then that function class is also PAC learnable [16, 20]. Two very recent works have studied the SQ hardness of learning data generated by quantum circuits. First, [21] analyze the hardness of learning the output distribution of clifford circuits and stabilizer states showing that these distributions are hard to learn using classical Boolean SQ oracles. Nevertheless, when given samples from the Boolean hypercube of the distribution, they provide an efficient algorithm based on linear regression to determine the stabilizer state underlying the distribution. Such a result is similar to classic results in [16] showing that parity functions are hard to learn using only SQ oracle calls but easy when performing linear regression with enough samples. Second, [22] show that learning stabilizer states is hard in an SQ setting where queries are made over two-outcome POVMs. Their results show that learning stabilizer states in such a setting is as hard as learning the function class of parity with noise in the standard Boolean setting. Our results expand the set of quantum functions that are hard to learn in SQ settings and relate such hardness results to the variational setting.

Supplementary Note 2.1– Quantum Statistical Query Models

Variational quantum algorithms are inherently noisy due to unavoidable sources such as the need for sampling outputs, or potentially correctable sources such as gate errors and state preparation noise. In such noisy settings, the statistical query (SQ) model provides a useful framework for quantifying the complexity of learning a class of functions by considering how many query calls to a noisy oracle are needed to learn any function in that class. As described in the main text, in the variational setting, we consider two forms of statistical queries which relate to learning a target Hamiltonian or a target unitary, both of which result in exponential hardness results for learning simple variational classes of data.

Our proofs expand on recent research showing hardness results in the SQ setting for certain quantum machine learning problems. More specifically, recent results that have shown that certain fundamental and rather simple classes of quantum "functions" are hard to learn in the SQ setting. Namely, (classical) output distributions of locally constructed quantum states [21] and the set of Clifford circuits [22] are hard to learn given properly chosen statistical query oracles. Following these results, we show that simple classes of functions generated by variational circuits are also exponentially difficult to learn in the SQ settings we consider. We also directly connect the statistical query setting to actual optimization algorithms that are used in practice for variational optimization. Our results indicate that training algorithms must be carefully constructed to avoid these poor lower bounds.

Supplementary Note 2.2– Limitations of Hardness Results in the SQ Framework

Though the SQ framework is a useful tool for analyzing the hardness of learning a class of functions in noisy settings, there are a few caveats and limitations of any hardness results proven in the SQ setting:

- The statistical query model inherently requires noise in the form of the tolerance τ. Furthermore, the guarantees of learning must handle worst case noise scenarios where the noise acts adversarially on the statistical query. Though quantum variational algorithms are inherently noisy, this noise typically does not arise in an adversarial nature.
- The statistical query model places bounds on learning classes of functions using optimizers that query this SQ model and is not directly related to issues of loss landscapes since there is no loss landscape to actually optimize. Nevertheless, since (noisy) calculations of gradients and loss function values are themselves examples of statistical queries, any issues with optimizing over a loss landscape will also arise in performing the optimizer through a series of statistical queries.
- Learning every function in a class $\mathcal{C}$ can be restrictive, and in practice, one may only really want to learn a given function or a small set of functions. In fact, it can be shown that even the class of functions generated by shallow neural networks is hard to learn in the SQ setting [23, 24, 25, 26, 27]; nevertheless, neural networks are very successful at learning specific functions such as the classification of real-world images [28].
- Specific to the settings considered here, our hardness results were obtained in the correlational SQ setting by constructing a family of orthogonal functions drawn from a given function class. We chose this setting for its close relation to the algorithms used in practice for performing optimization over variational parameters. However, as mentioned in the main text, the correlational SQ setting is strictly weaker than the more general SQ setting, and separations between SQ and correlational SQ results have been made in prior work [29, 30].

Supplementary Note 3– Proofs of Statistical Query Results

Throughout this section, we make use of standard formulas from Weingarten calculus to integrate over Haar measure or t-designs [31, 32, 33]. Let $|I_m^n\rangle$ denote n copies of the unnormalized maximally entangled state on a Hilbert space of dimension m:

$$|I_m^n\rangle = \sum_{i_1,i_2,\ldots,i_n=1}^{m} |i_1,i_2,\ldots,i_n\rangle\,|i_1,i_2,\ldots,i_n\rangle\,. \tag{15}$$

For $n=2$, let $\left|S_m^2\right\rangle$ denote the same unnormalized state as above with a swap operation applied to the second register:

$$\begin{aligned}\left|S_m^2\right\rangle &= (\mathbf{I}\otimes \mathrm{SWAP})\left|I_m^2\right\rangle \\ &= \sum_{i_1,i_2=1}^{m} |i_1,i_2\rangle\,|i_2,i_1\rangle\,.\end{aligned} \tag{16}$$

The following hold over a distribution $\mathcal{D}$ that is a 2-design over the unitary matrices of dimension m:

$$\begin{aligned}\mathbb{E}_{\mathbf{U}\sim\mathcal{D}}\left[\mathbf{U}\otimes\bar{\mathbf{U}}\right] &= \frac{1}{m}\left|I_m^1\right\rangle\left\langle I_m^1\right|, \\ \mathbb{E}_{\mathbf{U}\sim\mathcal{D}}\left[\mathbf{U}\otimes\mathbf{U}\otimes\bar{\mathbf{U}}\otimes\bar{\mathbf{U}}\right] &= \frac{1}{m^2-1}\left(\left|I_m^2\right\rangle\left\langle I_m^2\right| + \left|S_m^2\right\rangle\left\langle S_m^2\right|\right) - \frac{1}{m(m^2-1)}\left(\left|I_m^2\right\rangle\left\langle S_m^2\right| + \left|S_m^2\right\rangle\left\langle I_m^2\right|\right),\end{aligned} \tag{17}$$

where $\bar{\mathbf{U}}$ denotes the matrix with entries that are the complex conjugate of entries of $\mathbf{U}$.

As a simple example of applying the techniques above, we show that for unitaries $\mathbf{U}_*$ and $\mathbf{V}$ of dimension d^n (e.g., $d=2$ for qubits and n is the number of qubits), $\mathbb{E}_{\rho\sim\mathcal{D}}\left[\mathrm{Re}[\mathrm{Tr}\left(\mathbf{U}_*^\dagger\mathbf{V}\rho\right)]\right] = d^{-n}\,\mathrm{Re}[\mathrm{Tr}\left(\mathbf{U}_*^\dagger\mathbf{V}\right)]$ whenever $\mathcal{D}$ forms a 1-design. This is a crucial formula that we use in the evaluation of statistical queries to qUSQ.

Lemma 1. For any distribution $\mathcal{D}$ that is a 1-design over states of dimension d^n,

$$\mathbb{E}_{\rho\sim\mathcal{D}}\left[\mathrm{Re}[\mathrm{Tr}(\mathbf{W}^\dagger\mathbf{V}\rho)]\right] = d^{-n}\,\mathrm{Re}[\mathrm{Tr}(\mathbf{W}^\dagger\mathbf{V})]. \tag{18}$$

Proof. WLOG, we rewrite the equation above in terms of a distribution over pure states and with a slight abuse of notation, we let $\mathcal{D}$ also denote a distribution over unitary matrices $\mathbf{U}$ that forms a 1-design:

$$\mathbb{E}_{\rho\sim\mathcal{D}}\left[\mathrm{Re}[\mathrm{Tr}(\mathbf{W}^\dagger\mathbf{V}\rho)]\right] = \mathbb{E}_{\mathbf{U}\sim\mathcal{D}}\left[\mathrm{Re}[\langle 0|\,\mathbf{U}^\dagger\mathbf{W}^\dagger\mathbf{V}\mathbf{U}\,|0\rangle]\right]. \tag{19}$$

Using equation 15, we have:

$$\mathbb{E}_{\mathbf{U}\sim\mathcal{D}}\left[\mathrm{Re}[\langle 0|\,\mathbf{U}^\dagger\mathbf{W}^\dagger\mathbf{V}\mathbf{U}\,|0\rangle]\right] = \mathbb{E}_{\mathbf{U}\sim\mathcal{D}}\left[\langle I^1_{d^n}|\,\big((\mathbf{W}^\dagger\mathbf{V})\otimes\mathbf{I}\big)\big(\mathbf{U}\otimes\bar{\mathbf{U}}\big)\,|0\rangle\,|0\rangle\right]. \tag{20}$$

Applying equation 17, we have:

$$\begin{aligned}\mathbb{E}_{\mathbf{U}\sim\mathcal{D}}\left[\mathrm{Re}[\langle 0|\,\mathbf{U}^\dagger\mathbf{W}^\dagger\mathbf{V}\mathbf{U}\,|0\rangle]\right] &= \mathbb{E}_{\mathbf{U}\sim\mathcal{D}}\left[\mathrm{Re}[\langle I^1_{d^n}|\,\big((\mathbf{W}^\dagger\mathbf{V})\otimes\mathbf{I}\big)\big(\mathbf{U}\otimes\bar{\mathbf{U}}\big)\,|0\rangle\,|0\rangle]\right]\\ &= \frac{1}{d^n}\,\mathrm{Re}[\langle I^1_{d^n}|\,\big((\mathbf{W}^\dagger\mathbf{V})\otimes\mathbf{I}\big)\,|I^1_{d^n}\rangle\,\langle I^1_{d^n}|0\rangle\,|0\rangle]\\ &= \frac{1}{d^n}\,\mathrm{Re}[\mathrm{Tr}(\mathbf{W}^\dagger\mathbf{V})].\end{aligned} \tag{21}$$

Supplementary Note 3.1– Lower Bounds for Statistical Query Learning

For completeness, we include a proof of Theorem 3, copied below, which lower bounds the number of queries needed to learn a hypothesis class. qCSQ and qUSQ both take the form of an inner product so the proof holds for both cases. As a reminder we include the definitions of qCSQ and qUSQ below.

Quantum correlational statistical query (qCSQ) Given a target observable $\mathbf{M}$ and a distribution $\mathcal{D}$ of input states, a query $\mathrm{qCSQ}(\mathbf{O},\tau)$ takes in a bounded observable $\mathbf{O}$ with $\|\mathbf{O}\| \leq 1$ and a tolerance τ and returns a value in the range:

$$\mathbb{E}_{\rho\sim\mathcal{D}}\left[\mathrm{Tr}(\mathbf{O}\rho)\,\mathrm{Tr}(\mathbf{M}\rho) - \tau\right] \leq \mathrm{qCSQ}(\mathbf{O},\tau) \leq \mathbb{E}_{\rho\sim\mathcal{D}}\left[\mathrm{Tr}(\mathbf{O}\rho)\,\mathrm{Tr}(\mathbf{M}\rho) + \tau\right]. \tag{22}$$

Quantum unitary statistical query (qUSQ) Given a target unitary transformation $\mathbf{U}_*$ over a distribution $\mathcal{D}$ of inputs, the oracle $\mathrm{qUSQ}(\mathbf{V},\tau)$ takes in a unitary matrix $\mathbf{V}$ and a tolerance τ and returns a value in the range:

$$\mathbb{E}_{\rho\sim\mathcal{D}}\left[\mathrm{Re}[\mathrm{Tr}(\mathbf{U}_*^\dagger\mathbf{V}\rho)] - \tau\right] \leq \mathrm{qUSQ}(\mathbf{V},\tau) \leq \mathbb{E}_{\rho\sim\mathcal{D}}\left[\mathrm{Re}[\mathrm{Tr}(\mathbf{U}_*^\dagger\mathbf{V}\rho)] + \tau\right]. \tag{23}$$

Finally, we remind the reader of the definition of the statistical query dimension.

Definition 2 (Statistical query dimension [34, 20]). For a distribution $\mathcal{D}$ and concept class $\mathcal{H}$ where $\|\mathbf{M}\|^2_{\mathcal{D}} \leq C_{max}$ for all $\mathbf{M}\in\mathcal{H}$, the statistical query dimension ($\mathrm{SQ\text{-}DIM}_{\mathcal{D}}(\mathcal{H})$) is the largest positive integer d such that there exists d observables $\mathbf{M}_1, \mathbf{M}_2, \ldots, \mathbf{M}_d \in \mathcal{H}$ such that for all $i \neq j$: $|\langle \mathbf{M}_i, \mathbf{M}_j\rangle_{\mathcal{D}}| \leq C_{max}/d$.

From here, we provide a proof of the query complexity of learning a function class, similar to the proof in [18].

Theorem 3 (Query complexity of learning [18, 34]). Given a distribution $\mathcal{D}$ on inputs and a hypothesis class $\mathcal{H}$ where $\|\mathbf{M}\|^2_{\mathcal{D}} \leq C_{max}$ for all $\mathbf{M}\in\mathcal{H}$, let $d = \mathrm{SQ\text{-}DIM}_{\mathcal{D}}(\mathcal{H})$ be the statistical query dimension of $\mathcal{H}$. Any qCSQ or qUSQ learner making queries with tolerance $C_{max}\tau$ must make at least $(d\tau^2 - 1)/2$ queries to learn $\mathcal{H}$ up to error $C_{max}\tau$.

Proof. Since we are restricted to the weaker setting of correlational statistical queries in this study, we can reuse a simple and elegant proof from [18].

Let $\mathbf{M}_1, \mathbf{M}_2, \cdots, \mathbf{M}_d$ be d functions that saturate SQ-DIM$_{\mathcal{D}}(\mathcal{H})$, i.e., $\langle \mathbf{M}_i, \mathbf{M}_j \rangle_{\mathcal{D}} \leq 1/d$ for all $i \neq j$. Assume we apply query $\mathbf{O}$ and let $S = \{i \in [d] : \langle \mathbf{O}, \mathbf{M}_i \rangle_{\mathcal{D}} > C_{max}\tau\}$ Then, by simple application of Cauchy-Schwarz, we have that for any query $\mathbf{O}$:

$$\begin{aligned} \left\langle \mathbf{O}, \sum_{i \in S} \mathbf{M}_i \right\rangle_{\mathcal{D}}^2 &\leq C_{max} \left\| \sum_{i \in S} \mathbf{M}_i \right\|_{\mathcal{D}}^2 \\ &= C_{max} \sum_{i,j \in S} \langle \mathbf{M}_i, \mathbf{M}_j \rangle_{\mathcal{D}} \\ &\leq C_{max}^2 \left(|S| + |S|^2/d \right). \end{aligned} \tag{24}$$

Note, that we can also bound the quantity above from below by using the definition of S:

$$\left\langle \mathbf{O}, \sum_{i \in S} \mathbf{M}_i \right\rangle_{\mathcal{D}} \geq C_{max}|S|\tau. \tag{25}$$

Combining the above, we have that

$$|S| \leq d/(d\tau^2 - 1). \tag{26}$$

Similarly, defining $S' = \{i \in [d] : \langle \mathbf{O}, \mathbf{M}_i \rangle_{\mathcal{D}} < -C_{max}\tau\}$ with correlation less than $-\tau$, we follow the steps above to also note that $|S'| \leq d/(d\tau^2 - 1)$. Altogether, we have that $|S'| + |S| \leq 2d/(d\tau^2 - 1)$, which implies that each oracle call returning 0 is inconsistent with at most $2d/(d\tau^2 - 1)$ functions. This results in the lower bound stated, as d functions must be ruled inconsistent to learn the target class.

Supplementary Note 3.2– Proofs of Statistical Query Dimensions for Variational Function Classes

Proposition 4 (SQ dimension for $L = 1$ and fixed global measurement). Given n qubits, let $\mathcal{H}$ be the concept class containing functions $f : \mathbb{C}^{2^n} \to \mathbb{R}$ consisting of single qubit rotations followed by a global Pauli Z measurement, i.e. functions of the form

$$f(|\psi\rangle; \mathbf{U}_1, \mathbf{U}_2, \ldots, \mathbf{U}_n) = \langle\psi| \left(\mathbf{U}_1^\dagger \otimes \mathbf{U}_2^\dagger \otimes \cdots \otimes \mathbf{U}_n^\dagger \right) (\mathbf{Z}_1 \otimes \mathbf{Z}_2 \otimes \cdots \mathbf{Z}_n) (\mathbf{U}_1 \otimes \mathbf{U}_2 \otimes \cdots \otimes \mathbf{U}_n) |\psi\rangle, \tag{27}$$

where $|\psi\rangle$ is the input to the function and $\mathbf{U}_1, \mathbf{U}_2, \ldots, \mathbf{U}_n$ are the parameterized 1-qubit rotation operations on distinct qubits. Then, the concept class $\mathcal{H}$ has SQ dimension SQ-DIM$_{\mathcal{D}}(\mathcal{H}) \geq 3^n$ under any distribution of states that forms a 2-design.

Proof. The simple proof of this proposition relies on the fact that all Pauli operators are pairwise orthogonal for a 2-design, i.e. given two distinct Pauli operators $\mathbf{P}_1$ and $\mathbf{P}_2$ then $\mathbb{E}_{\rho\sim\mathcal{D}}[\mathrm{Tr}(\mathbf{P}_1\rho)\,\mathrm{Tr}(\mathbf{P}_2\rho)] = 0$. Therefore, we simply show that the concept class $\mathcal{H}$ is capable of producing any Pauli string not containing the identity.

To proceed, note that we can rewrite the function class as follows:

$$f(|\psi\rangle; \mathbf{U}_1, \mathbf{U}_2, \ldots, \mathbf{U}_n) = \langle\psi| (\mathbf{U}_1^\dagger \mathbf{Z}_1 \mathbf{U}_1) \otimes (\mathbf{U}_2^\dagger \mathbf{Z}_2 \mathbf{U}_2) \otimes \cdots \otimes (\mathbf{U}_n^\dagger \mathbf{Z}_n \mathbf{U}_n) |\psi\rangle. \tag{28}$$

To obtain any arbitrary Pauli string, we simply conjugate the $\mathbf{Z}_i$ operator for the i-th qubit by a corresponding operation. If the i-th qubit of a Pauli string is equal to $\mathbf{X}$, then we set $\mathbf{U}_i = \mathbf{H}$ or the Hadamard transform. Similarly, if the i-th qubit of a Pauli string is equal to $\mathbf{Y}$, then we set $\mathbf{U}_i = \mathbf{H}\sqrt{\mathbf{Z}}^\dagger$. By conjugation of the individual 1-qubit operators, we thus can produce any Pauli operator in $\{\mathbf{X}, \mathbf{Y}, \mathbf{Z}\}^{\otimes n}$.

Corollary 5. By application of Theorem 3, the class of functions defined in Proposition 4 consisting of a single layer of parameterized single qubit unitary gates and a fixed global measurement on n qubits requires $2^{\Omega(n)}$ queries to learn for a query tolerance greater than $3^{-\beta n}$, where $\beta = 1/2 - \Omega(1)$.

Proposition 6 (SQ dimension for $L = \lceil \log_2 n \rceil$, two-qubit gates, and single Pauli $\mathbf{Z}$ measurement). Given n qubits, let $\mathcal{H}$ be the concept class containing functions $f : \mathbb{C}^{2^n} \to \mathbb{R}$ consisting of $\lceil \log_2 n \rceil$ layers of two-qubit gates followed by a Pauli $\mathbf{Z}$ measurement on a single qubit. Then, the concept class $\mathcal{H}$ has SQ dimension $\text{SQ-DIM}_{\mathcal{D}}(\mathcal{H}) \geq 4^n - 1$ under any distribution of inputs that forms a 2-design.

Proof. We will show that $\mathcal{H}$ is powerful enough to perform any nontrivial Pauli measurement (i.e., any Pauli but the identity) and hence construct at least $4^n - 1$ orthogonal functions. Classically, any parity function can be constructed in $\lceil \log_2 n \rceil$ layers, and we use a similar construction here.

Without loss of generality, assume the Pauli measurement is on the first qubit. Let $\mathbf{U}(\theta)$ represent a possible unitary that can be applied using the given hypothesis class, resulting in a final measurement of $\mathbf{U}(\theta)^\dagger \mathbf{Z}_1 \mathbf{U}(\theta)$ on a given input state $|\psi\rangle$. We will show that we can parameterize the circuit such that for any Pauli measurement, $\mathbf{P}_1 \otimes \mathbf{P}_2 \otimes \cdots \otimes \mathbf{P}_n = \mathbf{U}(\theta)^\dagger \mathbf{Z}_1 \mathbf{U}(\theta)$ where $\mathbf{P}_i$ indicates the Pauli operator of qubit i (i.e., $\mathbf{P}_i \in \{\mathbf{I}, \mathbf{X}, \mathbf{Y}, \mathbf{Z}\}$).

To construct any Pauli operator $\mathbf{P}_1 \otimes \mathbf{P}_2 \otimes \cdots \otimes \mathbf{P}_n$, we follow the steps below:

1. In the first layer, apply a unitary to each qubit i which maps the computational basis to the basis of the Pauli for qubit i. In more detail, if $\mathbf{P}_i = \mathbf{I}$ or $\mathbf{P}_i = \mathbf{Z}$, then apply the identity map to keep the basis the same. If $\mathbf{P}_i = \mathbf{X}$, then apply the Hadamard transform and if $\mathbf{P}_i = \mathbf{Y}$ then apply the operation $\mathbf{H}\sqrt{\mathbf{Z}}^\dagger$.

2. In the l-th layer, apply a specific two qubit gate to qubit pairs $\{1, 2^{l-1} + 1\}, \{2(2^{l-1}) + 1, 3(2^{l-1}) + 1\}, \{4(2^{l-1}) + 1, 5(2^{l-1}) + 1\}, \ldots$. For a layer l and a given pair $\{i, j\}$, apply the following gate:

   - if all of $\mathbf{P}_i, \mathbf{P}_{i+1}, \ldots, \mathbf{P}_{j+2^{l-1}}$ are equal to $\mathbf{I}$, then apply the identity.
   - if any of $\mathbf{P}_i, \mathbf{P}_{i+1}, \ldots, \mathbf{P}_{j-1}$ are not equal to $\mathbf{I}$ and all of $\mathbf{P}_j, \mathbf{P}_{j+1}, \ldots, \mathbf{P}_{j+2^{l-1}}$ are equal to $\mathbf{I}$ then apply the identity as well.
   - if all of $\mathbf{P}_i, \mathbf{P}_{i+1}, \ldots, \mathbf{P}_{j-1}$ are equal to $\mathbf{I}$ and any of $\mathbf{P}_j, \mathbf{P}_{j+1}, \ldots, \mathbf{P}_{j+2^{l-1}}$ are not equal to $\mathbf{I}$, then apply a swap gate between qubits i and j.
   - otherwise, apply the following 2-qubit gate to i and j which conjugates $\mathbf{Z} \otimes \mathbf{I}$ to $\mathbf{Z} \otimes \mathbf{Z}$:

$$\begin{bmatrix} 1 & 0 & 0 & 0 \\ 0 & 0 & 0 & 1 \\ 0 & 0 & 1 & 0 \\ 0 & 1 & 0 & 0 \end{bmatrix}. \tag{29}$$

3. repeat step 2 above starting from $l = 1$ to $l = \lceil \log_2 n \rceil$. Measuring the first qubit will measure the corresponding desired Pauli. Note, that the single qubit operations of step 1 and the two-qubit operations of step 2 can be combined into a single 2-qubit gate thus not changing the depth.

Following the steps above, at layer l, the measurement of the first qubit corresponds to the Pauli measurement of the first 2^l qubits. Recursively applying this procedure l layers produces any arbitrary Pauli string.

Corollary 7. By application of Theorem 3, the class of functions defined in Proposition 6 consisting of $\lceil \log_2 n \rceil$ two-qubit unitary gates and a fixed measurement on a single qubit requires $2^{\Omega(n)}$ queries to learn for a query tolerance greater than $4^{-\beta n}$, where $\beta = 1/2 - \Omega(1)$.

Proposition 8 (SQ dimension for L layers, neighboring 2-local gates in one-dimensional lattice and fixed single qubit measurement). Given n qubits, let $\mathcal{H}$ be the concept class containing functions $f : \mathbb{C}^{2^n} \to \mathbb{R}$ consisting of L layers of 2-qubit unitary operations followed by a Pauli Z measurement on a single qubit (labeled qubit m), i.e. functions of the form

$$f(|\psi\rangle; \mathbf{W}_1, \mathbf{W}_2, \ldots, \mathbf{W}_L) = \langle\psi| \mathbf{W}_1^\dagger \mathbf{W}_2^\dagger \cdots \mathbf{W}_L^\dagger (\mathbf{Z}_m) \mathbf{W}_L \cdots \mathbf{W}_2 \mathbf{W}_1 |\psi\rangle, \tag{30}$$

where $|\psi\rangle$ is the input to the function and $\mathbf{W}_1, \mathbf{W}_2, \ldots, \mathbf{W}_L$ are the unitary operations at each layer consisting of tensor products of 2-local unitary operators acting on neighboring qubits. Then, the concept class $\mathcal{H}$ has SQ dimension $\text{SQ-DIM}_{\mathcal{D}}(\mathcal{H}) \geq 4^{\min(2L,n)} - 1$ under any distribution of states that forms a 2-design.

Proof. Our proof relies on the fact that with L layers, one can conjugate the fixed single qubit measurement on qubit m to produce any $2L$-qubit Pauli on the $2L$ qubits within the reverse light cone of m. We follow a proof outline similar to Proposition 6.

Before we proceed, we assume without loss of generality, that L is odd and the first layer applies a two qubit unitary to qubit m and the preceeding qubit $m-1$. It is straightforward to extend this to the case where L is even. Therefore, qubit m is the L-th qubit in the reverse light cone of qubit m, i.e., the light cone traverses qubits $m-L$ to $m+L-1$. For the steps below, we then index the qubits from $-L$ to $L-1$ so that the numbering is relative to qubit m. To perform a given $2L$ Pauli operator $\mathbf{P}_{-L} \otimes \mathbf{P}_{-L+1} \otimes \cdots \otimes \mathbf{P}_{L-1}$ in the reverse light cone of qubit m, we follow the steps below, many of which are copied from Proposition 6.:

1. In the first layer, apply a unitary to each qubit i which maps the computational basis to the basis of the Pauli for qubit i. In more detail, if $\mathbf{P}_i = \mathbf{I}$ or $\mathbf{P}_i = \mathbf{Z}$, then apply the identity map to keep the basis the same. If $\mathbf{P}_i = \mathbf{X}$, then apply the Hadamard transform and if $\mathbf{P}_i = \mathbf{Y}$ then apply the operation $\mathbf{H}\sqrt{\mathbf{Z}}^\dagger$.

2. in the L-th layer, for the 2-qubit unitary acting on qubits 0 and -1, apply the following gate:
   - if all of $\mathbf{P}_{-L}, \mathbf{P}_{-L+1}, \ldots, \mathbf{P}_{-1}$ are equal to $\mathbf{I}$ and all of $\mathbf{P}_1, \mathbf{P}_2, \ldots, \mathbf{P}_{L-1}$ are equal to $\mathbf{I}$, then apply the identity.
   - if any of $\mathbf{P}_{-L}, \mathbf{P}_{-L+1}, \ldots, \mathbf{P}_{-1}$ are not equal to $\mathbf{I}$ and and any of $\mathbf{P}_1, \mathbf{P}_2, \ldots, \mathbf{P}_{L-1}$ are not equal to $\mathbf{I}$, apply the following 2-qubit gate (CNOT) to i and $i+1$ which conjugates $\mathbf{I} \otimes \mathbf{Z}$ to $\mathbf{Z} \otimes \mathbf{Z}$:
   $$\begin{bmatrix} 1 & 0 & 0 & 0 \\ 0 & 1 & 0 & 0 \\ 0 & 0 & 0 & 1 \\ 0 & 0 & 1 & 0 \end{bmatrix}. \tag{31}$$
   - otherwise, apply the swap operation between qubits 0 and -1.

3. In the l-th layer for any $l \neq L$, apply a specific two qubit gate to neighboring qubit pairs $\{-L+l-1, -L+l\}$ on the edge of the reverse light cone. For simplicity, let $i = -L+1-1$ and apply the following gate to qubit pair $\{i, i+1\}$:
   - if all of $\mathbf{P}_{-L}, \mathbf{P}_{-L+1}, \ldots, \mathbf{P}_i$ are equal to $\mathbf{I}$, then apply the identity.
   - if any of $\mathbf{P}_{-L}, \mathbf{P}_{-L+1}, \ldots, \mathbf{P}_i$ are not equal to $\mathbf{I}$ and $\mathbf{P}_{i+1} = \mathbf{I}$, then apply a swap between qubits i and $i+1$.
   - otherwise, apply the following 2-qubit gate (CNOT) to i and $i+1$ which conjugates $\mathbf{I} \otimes \mathbf{Z}$ to $\mathbf{Z} \otimes \mathbf{Z}$:
   $$\begin{bmatrix} 1 & 0 & 0 & 0 \\ 0 & 1 & 0 & 0 \\ 0 & 0 & 0 & 1 \\ 0 & 0 & 1 & 0 \end{bmatrix}. \tag{32}$$

   Similarly, for the other edge of the reverse light cone, we apply the same gates, but in "reverse" logic. Here, we apply a 2-qubit unitary to qubit pair $\{L-l-2, L-l-1\}$. For simplicity, let $i = L-l-2$ and apply the following gate to qubit pair $\{i, i+1\}$:
   - if all of $\mathbf{P}_{i+1}, \mathbf{P}_{i+2}, \ldots, \mathbf{P}_{L-1}$ are equal to $\mathbf{I}$, then apply the identity.
   - if any of $\mathbf{P}_{i+1}, \mathbf{P}_{i+2}, \ldots, \mathbf{P}_{L-1}$ are not equal to $\mathbf{I}$ and $\mathbf{P}_{i+1} = \mathbf{I}$, then apply a swap between qubits i and $i+1$.

- otherwise, apply the following 2-qubit gate to i and $i+1$ which conjugates $\mathbf{Z} \otimes \mathbf{I}$ to $\mathbf{Z} \otimes \mathbf{Z}$:

$$\begin{bmatrix} 1 & 0 & 0 & 0 \\ 0 & 0 & 0 & 1 \\ 0 & 0 & 1 & 0 \\ 0 & 1 & 0 & 0 \end{bmatrix}. \tag{33}$$

4. repeat step 3 above starting from $l = 1$ to $l = L - 1$. Measuring the m-th qubit will measure the corresponding desired Pauli. Note, that the single qubit operations of step 1 and the two-qubit operations of step 2 can be combined into a single 2-qubit gate thus not changing the depth.

Corollary 9. By application of Theorem 3, the class of functions defined in Proposition 8 consisting of L layers of neighboring 2-qubit gates and a fixed measurement on a single qubit requires $2^{\Omega(\min(2L,n))}$ queries to learn for a constant query tolerance that does not depend on L and n.

The above can be generalized to lower bound the statistical query dimension for circuits of L layers on d-dimensional lattices as we show below. In d-dimensional lattices, since the light cone of a single qubit measurement grows at a rate of L^d for an L layers, we can prove that the statistical query dimension grows as $2^{\Omega\left(\min\left(2L,n^{1/d}\right)^d\right)}$.

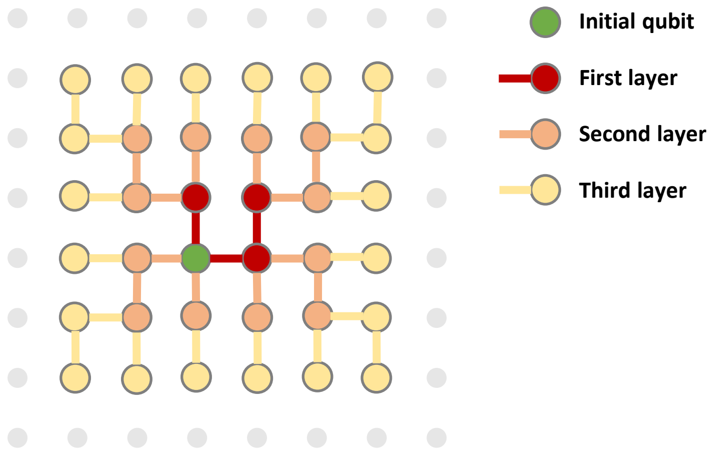

Supplementary Fig. 1: **Light cone growth.** Growth of the light cone for a 2-dimensional lattice, where the initial qubit is the one that is measured. The size of the lattice grows with the perimeter of the light cone for each layer which consists of local 2-qubit gates applied in each dimension. Each qubit is connected to a qubit in the edge of the light cone of the prior layer, forming a graph which is a tree rooted at the initial qubit.

Proposition 10 (SQ dimension for L layers, neighboring 2-local gates on d-dimensional lattice and fixed single qubit measurement). Given n qubits, let $\mathcal{H}$ be the concept class containing functions $f : \mathbb{C}^{2^n} \to \mathbb{R}$ consisting of L layers of 2-qubit unitary operations applied in each dimension followed by a Pauli Z measurement on a single qubit (labeled qubit m), i.e. functions of the form

$$f(|\psi\rangle ; \mathbf{W}_1, \mathbf{W}_2, \ldots, \mathbf{W}_L) = \langle\psi| \mathbf{W}_1^\dagger \mathbf{W}_2^\dagger \cdots \mathbf{W}_L^\dagger (\mathbf{Z}_m) \mathbf{W}_L \cdots \mathbf{W}_2 \mathbf{W}_1 |\psi\rangle, \tag{34}$$

where $|\psi\rangle$ is the input to the function and $\mathbf{W}_1, \mathbf{W}_2, \ldots, \mathbf{W}_L$ are the unitary operations at each layer consisting of tensor products of 2-local unitary operators acting along each dimension on neighboring qubits in a d-dimensional lattice. Then, the concept class $\mathcal{H}$ has SQ dimension $\text{SQ-DIM}_{\mathcal{D}}(\mathcal{H}) = 2^{\Omega\left(\min\left(2L, n^{1/d}\right)^d\right)}$ under any distribution of states that forms a 2-design.

Proof. Our proof relies on the fact that with L layers, one can conjugate the fixed single qubit measurement on qubit m to produce any Pauli on the $\Omega(L^d)$ qubits within the reverse light cone of m. We follow a proof outline similar to Proposition 8.

To be more precise, let us introduce some notation. To perform any Pauli measurement in the reverse light cone at a given layer $l \in [L]$ indexed in reverse order, we apply gates to the perimeter of the reverse light cone at layer $l-1$. We assume there are N_l qubits in the reverse light cone at layer l and index these qubits from 1 to N_l to construct the Pauli $\mathbf{P}_1 \otimes \mathbf{P}_2 \otimes \cdots \otimes \mathbf{P}_{N_l}$. Like in Proposition 8, we grow the Pauli at each layer.

To grow the light cone and properly choose the 2-qubit gates, we construct a graph which is a tree where the parent of any qubit is the prior qubit which it was connected to in the light cone of the previous layer (see Supplementary Fig. 1 for an example). The root of the tree is the qubit which is being measured. For example, at layer $l = 1$, the light cone is of size two in each dimension and the qubit being measured is the parent to the child node which it is connected to. To construct any pauli $\mathbf{P}_1 \otimes \mathbf{P}_2 \otimes \cdots \otimes \mathbf{P}_{N_L}$, we follow the steps below:

1. in the l-th layer, for all parent and child qubits p and c respectively connected in the tree at layer l, apply a unitary acting on qubits p and c as follows:
   - if all of the qubits that are descendants of qubit c and qubit c itself have Pauli terms that are equal to $\mathbf{I}$, then apply the identity gate between qubit p and c.
   - if any of the qubits that are descendants of qubit c or qubit c itself have Pauli terms that are not equal to $\mathbf{I}$ and the Pauli term of qubit p is equal to $\mathbf{I}$, then apply the swap gate between p and c.
   - otherwise, apply the following 2-qubit gate to qubits p and c which conjugates $\mathbf{Z} \otimes \mathbf{I}$ to $\mathbf{Z} \otimes \mathbf{Z}$:

$$\begin{bmatrix} 1 & 0 & 0 & 0 \\ 0 & 0 & 0 & 1 \\ 0 & 0 & 1 & 0 \\ 0 & 1 & 0 & 0 \end{bmatrix}. \tag{35}$$

2. repeat the step above starting from $l = 1$ to $l = L$.
3. In the first layer ($l = L$), apply a unitary to each qubit i which maps the computational basis to the basis of the Pauli for qubit i. In more detail, if $\mathbf{P}_i = \mathbf{I}$ or $\mathbf{P}_i = \mathbf{Z}$, then apply the identity map to keep the basis the same. If $\mathbf{P}_i = \mathbf{X}$, then apply the Hadamard transform and if $\mathbf{P}_i = \mathbf{Y}$ then apply the operation $\mathbf{H}\sqrt{\mathbf{Z}}^\dagger$.

Following the above steps, measuring the single qubit will measure the corresponding desired Pauli. Note, that the single qubit operations of the first layer and the two-qubit operations of that layer can be combined into a single 2-qubit gate thus not changing the depth.

In each layer, 2-qubit gates act along each dimension in some order. We can assume an ordering of the dimensions without loss of generality and assume that we apply gates along that dimension in order. After the first layer, the lattice has size 2 along each dimension. For each layer thereafter, the lattice grows by 2 qubits in each dimension (see Supplementary Fig. 1 for example). Therefore, the reverse light cone grows at a rate $\Omega(L^d)$. Since the light cone can be at most of size n (number of qubits), then the light cone is of size $2^{\Omega\left(\min\left(2L, n^{1/d}\right)^d\right)}$ for all L layers.

Corollary 11. By application of Theorem 3, the class of functions defined in Proposition 10 consisting of L layers of neighboring 2-qubit gates and a fixed measurement on a single qubit requires $2^{\Omega\left(\min\left(2L, n^{1/d}\right)^d\right)}$ queries to learn for a query tolerance that decays no faster than $2^{\omega\left(\min\left(2L, n^{1/d}\right)^d\right)}$. For $L \ll n$, this is equal to $2^{\Omega(L^d)}$ for any constant query tolerance that does not depend on L or n.

Proposition 12 (SQ dimension for $L = 1$, unitary compiling, and single qubit gates). Given n qubits, let $\mathcal{H}$ be the concept class containing unitary transformations $\mathbf{V} : \mathbb{C}^{2^n} \to \mathbb{C}^{2^n}$ consisting of single qubit rotations in a single layer

$$\mathbf{V}(|\psi\rangle, \mathbf{U}_1, \mathbf{U}_2, \ldots, \mathbf{U}_n) = \mathbf{U}_1 \otimes \mathbf{U}_2 \otimes \cdots \otimes \mathbf{U}_n |\psi\rangle, \tag{36}$$

where $|\psi\rangle$ is the input to the transformation and $\mathbf{U}_1, \mathbf{U}_2, \ldots, \mathbf{U}_n$ are the parameterized 1-qubit operations. Then, the concept class $\mathcal{H}$ has SQ dimension $\text{SQ-DIM}_{\mathcal{D}}(\mathcal{H}) \geq 4^n$ under the qUSQ model and any distribution $\mathcal{D}$ of inputs that is a 2-design.

Proof. From Lemma 1, we have that $\langle \mathbf{U}, \mathbf{V} \rangle_{\mathcal{D}} = 2^{-n} \operatorname{Re}\left[\operatorname{Tr}\left(\mathbf{U}^\dagger \mathbf{V}\right)\right]$. With one layer of single qubit unitary operations, any Pauli matrix can be constructed. Since $\operatorname{Tr}(\mathbf{P}_1 \mathbf{P}_2) = 0$ for any two distinct Pauli matrices $\mathbf{P}_1$ and $\mathbf{P}_2$, there are at least 4^n matrices in $\mathcal{H}$ which are orthogonal under the inner product.

Corollary 13. By application of Theorem 3, the class of functions defined in Proposition 12 consisting of a single layer of single qubit unitaries requires $2^{\Omega(n)}$ queries to learn for a query tolerance greater than $4^{-\beta n}$, where $\beta < 1/2 - \Omega(1)$.

Supplementary Note 3.3– Swap Test via Statistical Queries

In the task of unitary compiling, one is given copies of states which are inputs and outputs of a target unitary transformation, and the goal is to learn the unitary transformation from those states. More formally, we aim to learn a unitary $\mathbf{U}_*$ given a distribution over inputs or a dataset of m state pairs $\{|\phi_i\rangle, \mathbf{U}_* |\phi_i\rangle\}_{i \in [m]}$.

One means of measuring overlaps between states is via the swap test [35]. For pure states, $|\phi\rangle$ and $|\psi\rangle$, the swap test measures the fidelity $|\langle\phi|\psi\rangle|^2$. The measured register in the swap test outputs $|0\rangle$ with probability $1/2 + |\langle\phi|\psi\rangle|^2/2$ and $|1\rangle$ otherwise. As we show in the main text, this quantity can be calculated using queries to qUSQ. We use the helper lemma below to prove this fact.

Lemma 14. For any distribution $\mathcal{D}$ that is a 2-design on a Hilbert space of dimension m,

$$\mathbb{E}_{\rho \sim \mathcal{D}}\left[\operatorname{Tr}\left(\mathbf{U}_* \rho \mathbf{U}_*^\dagger \mathbf{V} \rho \mathbf{V}^\dagger\right)\right] = \frac{m^{-1}\left|\operatorname{Tr}\left(\mathbf{V}^\dagger \mathbf{U}_*\right)\right|^2 + 1}{m+1}. \tag{37}$$

Proof. WLOG, we rewrite the equation above in terms of a distribution over pure states. Since mixed states are themselves distributions over pure states, this can always be done. Therefore, with a slight abuse of notation, we let $\mathcal{D}$ also denote a distribution over unitary matrices $\mathbf{U}$ that forms a 2-design:

$$\begin{aligned}\mathbb{E}_{\rho \sim \mathcal{D}}\left[\operatorname{Tr}\left(\mathbf{U}_* \rho \mathbf{U}_*^\dagger \mathbf{V} \rho \mathbf{V}^\dagger\right)\right] &= \mathbb{E}_{\mathbf{U} \sim \mathcal{D}}\left[\langle 0| \mathbf{U}^\dagger \mathbf{V}^\dagger \mathbf{U}_* \mathbf{U} |0\rangle \langle 0| \mathbf{U}^\dagger \mathbf{U}_*^\dagger \mathbf{V} \mathbf{U} |0\rangle\right] \\ &= \mathbb{E}_{\mathbf{U} \sim \mathcal{D}}\left[\left|\langle 0| \mathbf{U}^\dagger \mathbf{V}^\dagger \mathbf{U}_* \mathbf{U} |0\rangle\right|^2\right].\end{aligned} \tag{38}$$

Using equation 15 and equation 16, we have

$$\begin{aligned}\mathbb{E}_{\mathbf{U} \sim \mathcal{D}}\left[\left|\langle 0| \mathbf{U}^\dagger \mathbf{V}^\dagger \mathbf{U}_* \mathbf{U} |0\rangle\right|^2\right] &= \mathbb{E}_{\mathbf{U} \sim \mathcal{D}}\left[\langle 0| \mathbf{U}^\dagger \mathbf{V}^\dagger \mathbf{U}_* \mathbf{U} |0\rangle \langle 0| \mathbf{U}^\dagger \mathbf{U}_*^\dagger \mathbf{V} \mathbf{U} |0\rangle\right] \\ &= \mathbb{E}_{\mathbf{U} \sim \mathcal{D}}\left[\left\langle I_m^2\right| \left((\mathbf{V}^\dagger \mathbf{U}_*) \otimes (\mathbf{U}_*^\dagger \mathbf{V}) \otimes \mathbf{I} \otimes \mathbf{I}\right)\left(\mathbf{U} \otimes \mathbf{U} \otimes \bar{\mathbf{U}} \otimes \bar{\mathbf{U}}\right) |0\rangle^{\otimes 4}\right].\end{aligned} \tag{39}$$

Applying equation 17, we have

$$\begin{aligned}
\mathbb{E}_{\mathbf{U}\sim\mathcal{D}}\left[\left|\langle 0|\,\mathbf{U}^\dagger\mathbf{V}^\dagger\mathbf{U}_*\mathbf{U}\,|0\rangle\right|^2\right] &= \frac{1}{m^2-1}\left\langle I_m^2\right|\left((\mathbf{V}^\dagger\mathbf{U}_*)\otimes(\mathbf{U}_*^\dagger\mathbf{V})\otimes\mathbf{I}\otimes\mathbf{I}\right)\left(\left|I_m^2\right\rangle\left\langle I_m^2\right| + \left|S_m^2\right\rangle\left\langle S_m^2\right|\right)|0\rangle^{\otimes 4} \\
&\quad - \frac{1}{m(m^2-1)}\left\langle I_m^2\right|\left((\mathbf{V}^\dagger\mathbf{U}_*)\otimes(\mathbf{U}_*^\dagger\mathbf{V})\otimes\mathbf{I}\otimes\mathbf{I}\right)\left(\left|I_m^2\right\rangle\left\langle S_m^2\right| + \left|S_m^2\right\rangle\left\langle I_m^2\right|\right)|0\rangle^{\otimes 4} \\
&= \frac{1}{m^2-1}\left(\mathrm{Tr}\left[\mathbf{V}^\dagger\mathbf{U}_*\right]\mathrm{Tr}\left[\mathbf{U}_*^\dagger\mathbf{V}\right] + \mathrm{Tr}\left[\mathbf{V}^\dagger\mathbf{U}_*\mathbf{U}_*^\dagger\mathbf{V}\right]\right) \\
&\quad - \frac{1}{m(m^2-1)}\left(\mathrm{Tr}\left[\mathbf{V}^\dagger\mathbf{U}_*\right]\mathrm{Tr}\left[\mathbf{U}_*^\dagger\mathbf{V}\right] + \mathrm{Tr}\left[\mathbf{V}^\dagger\mathbf{U}_*\mathbf{U}_*^\dagger\mathbf{V}\right]\right) \\
&= \left(\frac{1}{m^2-1} - \frac{1}{m(m^2-1)}\right)\left(\left|\mathrm{Tr}\left[\mathbf{V}^\dagger\mathbf{U}_*\right]\right|^2 + m\right) \\
&= \frac{m^{-1}\left|\mathrm{Tr}\left[\mathbf{V}^\dagger\mathbf{U}_*\right]\right|^2 + 1}{m+1}.
\end{aligned} \tag{40}$$

Supplementary Note 4– Shallow VQAs as Random Fields

Supplementary Note 4.1– Random Fields on Manifolds

Hardness results from barren plateaus or SQ models both intuitively arise from the exponential decay of quantities necessary to perform optimization. To analyze the shallow circuit setting beyond the SQ model—where such exponentially decaying quantities tend not to exist—we look toward models of variational loss landscapes as random fields on manifolds. This mirrors [36, 37, 12] in studying the loss landscapes of machine learning models via mapping to certain random fields which are easier to study analytically. As in [12], here we show that certain classes of variational loss functions of shallow quantum models converge in some limit to Wishart hypertoroidal random fields (WHRFs), for which results on the loss landscape are already known [12]. We give a brief review here.

WHRFs in q variables are random fields on a specific tensor product embedding of the hypertorus $\left(S^1\right)^{\times q}$ in $\mathbb{R}^{2^q}$. More specifically, points on this embedding are described by the Kronecker product:

$$\mathbf{w} = \bigotimes_{i=1}^{q}\begin{pmatrix}\cos\left(\theta_i\right)\\ \sin\left(\theta_i\right)\end{pmatrix} \tag{41}$$

for angles $-\pi \leq \theta_i < \pi$. These random fields are then of the form:

$$F_{\mathrm{WHRF}}\left(\boldsymbol{\theta}\right) = \mathbf{w}^\intercal\cdot\mathbf{J}\cdot\mathbf{w}, \tag{42}$$

where $\mathbf{J}$ is drawn from the normalized complex Wishart distribution $\mathcal{CW}_{2^q}\left(m,\mathbf{\Sigma}\right)$ with m degrees of freedom. The complex Wishart distribution is a natural multivariate generalization of the gamma distribution, and is given by the distribution of the square of a complex Gaussian random matrix. Specifically, for $\mathbf{X}\in\mathbb{C}^{N\times m}$ a matrix with i.i.d. complex Gaussian columns with covariance matrix $\mathbf{\Sigma}$, the matrix

$$\mathbf{W} = \frac{1}{m}\mathbf{X}\cdot\mathbf{X}^\dagger \tag{43}$$

is normalized complex Wishart distributed with scale matrix $\mathbf{\Sigma}$ and m degrees of freedom. As discussed in [12], the loss landscapes of WHRFs exhibit a complexity phase transition governed by the overparameterization ratio

$$\gamma = \frac{q}{2m}, \tag{44}$$

where models with $\gamma \geq 1$ have local minima near the global minimum, and models with $\gamma \ll 1$ have local minima far from the global minimum. Thus, the degrees of freedom parameter m plays a pivotal role in governing the loss landscapes of WHRFs: when q is much smaller than m, training is typically infeasible due to an abundance of "traps" in the training landscape. Our main result here is in demonstrating that even for certain shallow VQAs, the corresponding WHRF is such that $\gamma \ll 1$, and training is infeasible.

Supplementary Note 4.2– Shallow VQAs Converge in Distribution to WHRFs

As discussed informally in the main text, our goal is to demonstrate that certain distributions of shallow variational quantum algorithms (VQAs) weakly converge to Wishart hypertoroidal random fields (WHRFs). The distribution of local minima of WHRFs was shown in [12] to exhibit a phase transition in trainability, where underparameterized models are untrainable due to poor local minima, and overparameterized models exhibit local minima close to the global minimum (though may still be untrainable for other reasons, e.g. due to barren plateaus [38, 39, 31]).

Unlike the nonlocal ansatz case [12], here we are unable to show the full convergence in distribution of shallow local VQAs to WHRFs. Instead, we focus on the joint distribution of the loss function, gradient norm, and Hessian determinant, where the gradient and Hessian have been normalized by the number of parameters q in the reverse light cone of each term in the Pauli expansion of the problem Hamiltonian; by the parameter shift rule [40, 41], it is easy to see that this bounds the gradient norm and Hessian eigenvalues as q is large. The local minima results of [12] depend only on this joint distribution, and thus showing this convergence suffices for our purposes.

We now review the setup of the VQA loss functions we are considering. Throughout the course of this review, we will make various assumptions, particularly on the distribution of gates in the VQA ansatz and on the independence of various reverse light cones; we discuss these assumptions and whether or not they are reasonable in more detail at the end of this Section. As mentioned in the main text, we consider optimizing VQAs on the problem Hamiltonian $\mathbf{H} \neq \mathbf{0}$, which has Pauli decomposition:

$$\mathbf{H} = \sum_{i=1}^{A} \alpha_i \mathbf{P_i}. \tag{45}$$

WLOG, we assume here $\mathbf{H}$ is traceless, and that all $\alpha_i > 0$. To simplify our analysis, we will consider the case where the reverse light cone of each term $\alpha_i \mathbf{P_i}$ in the Pauli decomposition of $\mathbf{H}$ is i.i.d. drawn from the same distribution of ansatzes, with the same parameter dependence. To make this more concrete, assume that the reverse light cone of each $\alpha_i \mathbf{P_i}$ is of the form $\mathbf{V_i}(\boldsymbol{\theta})\left|\mathbf{0}\right\rangle$ where $\boldsymbol{\theta} \in \mathbb{R}^q$, and has support on a number $l \ll n$ of qubits. In this regime, we can scale and shift the loss landscape of the standard variational loss function

$$F(\boldsymbol{\theta}) = \left\langle\boldsymbol{\theta}\right| \mathbf{H} \left|\boldsymbol{\theta}\right\rangle \tag{46}$$

to be of the form:

$$F_{\text{VQE}}(\boldsymbol{\theta}) = 1 - \lambda_0^{-1} \sum_{i=1}^{A} \alpha_i \left\langle\mathbf{0}\right| \mathbf{V_i}(\boldsymbol{\theta})^\dagger \mathbf{P_i} \mathbf{V_i}(\boldsymbol{\theta}) \left|\mathbf{0}\right\rangle = 1 + \left\|\boldsymbol{\alpha}\right\|_1^{-1} \sum_{i=1}^{A} \alpha_i \left\langle\mathbf{0}\right| \mathbf{V_i}^\dagger(\boldsymbol{\theta}) \mathbf{P_i} \mathbf{V_i}(\boldsymbol{\theta}) \left|\mathbf{0}\right\rangle, \tag{47}$$

where λ_0 is the ground state energy of $\mathbf{H}$ and $\boldsymbol{\alpha}$ is the vector of all α_i.

We assume that $\mathbf{V_i}(\boldsymbol{\theta})$ is of the form:

$$\mathbf{V_i}(\boldsymbol{\theta}) = \mathbf{W_i}(\boldsymbol{\theta}) \mathbf{U_i}, \tag{48}$$

where $\mathbf{U_i}$ are i.i.d. drawn from an ϵ-approximate t-design under the monomial measure on l qubits [42], where $\epsilon = \mathrm{O}(1)$. Note that in particular, though the total ansatz size n may be large, all potential scrambling of the ansatz may only happen locally, in regions of size $l \ll n$; in other words, these ansatzes are not expected to suffer from barren plateaus, particularly if $l = \mathrm{O}(\log(n))$ [39, 31]. $\mathbf{W_i}$ is composed of fixed parameterized rotations which we take WLOG to be of the form $\mathbf{R_{Y_a}}(\theta_b) = \exp(-\mathrm{i}\theta_b \mathbf{Y_a})$ (where as previously mentioned,

this parameter dependence is identical across all $\mathbf{W_i}$), fixed gates, and potentially randomly chosen gates such that $\mathbf{W_i}$ itself is a random field. For simplicity, we also assume that all θ_i are independent from one another (i.e. we are in the $r = 1$ regime of [12]), and that each qubit in the reverse light cone has at least one parameterized gate. We also assume that the field $\mathbf{W_i}(\boldsymbol{\theta})$ is rotationally invariant in θ_i.

We now give the formal statement and proof of the loss landscapes of local, shallow VQAs. First, the formal statement:

Theorem 15 (Approximately locally scrambled variational loss functions converge to WHRFs). Let $p_{\mathrm{VQE},\boldsymbol{\theta}}$ be the joint distribution of the loss function of equation 47, its gradient norm, and the determinant of its Hessian at $\boldsymbol{\theta}$, where the gradient and Hessian are normalized by q. Let $p_{\mathrm{WHRF},\boldsymbol{\theta}}$ be the same for the WHRF:

$$F_{\mathrm{WHRF}}(\boldsymbol{\theta}) = m^{-1} \sum_{i,j=1}^{2^l} w_i J_{i,j} w_j \tag{49}$$

with $m = \frac{\|\boldsymbol{\alpha}\|_1^2}{\|\boldsymbol{\alpha}\|_2^2} 2^{l-1}$ degrees of freedom, where $\mathbf{J} \sim \mathcal{CW}_{2^l}(m, \mathbf{I_{2^l}})$. Here, $\mathbf{w}$ are points on the hypertorus $\left(S^1\right)^{\times l}$ parameterized by $\tilde{\boldsymbol{\theta}}$, where $\tilde{\theta}_i$ is the sum of all θ_j on qubit i. We then have that $p_{\mathrm{VQE},\boldsymbol{\theta}}$ weakly converges to $p_{\mathrm{WHRF},\boldsymbol{\theta}}$, up to an error $\tilde{\mathrm{O}}\left(\mathrm{poly}\left(\frac{1}{t} + \epsilon + \exp(-l)\right)\right)$ in Lévy–Prokhorov distance.

As we previously mentioned, for technical reasons, we only prove the convergence of the joint distribution of the loss and certain functions of its first two derivatives. We emphasize once more that this does not affect our final conclusions, as all results on the local minima distribution of WHRFs given in [12] depend only on this joint distribution.

To prove Theorem 15, we begin by showing that, up to terms that go to zero polynomially quickly as $\epsilon \to 0, t \to \infty$, one can WLOG consider ansatzes of the form of equation 47 that are explicitly Haar random within each reverse light cone of size l.

Lemma 16 (Approximate local scrambling bound on the loss function and its derivatives). Let $p_{\mathrm{VQE},\boldsymbol{\theta}}$ be the joint distribution described in Theorem 15. Let $p_{\mathrm{Haar},\boldsymbol{\theta}}$ be the same, for $\mathbf{U_i}$ taken to be i.i.d. Haar random. We then have that $p_{\mathrm{VQE},\boldsymbol{\theta}}$ weakly converges to $p_{\mathrm{Haar},\boldsymbol{\theta}}$, up to an error $\tilde{\mathrm{O}}\left(\mathrm{poly}\left(\frac{1}{t} + \epsilon\right)\right)$ in Lévy–Prokhorov distance.

Proof. Let $\phi_{\mathrm{VQE}}(\mathbf{x} \mid \boldsymbol{\theta})$ be the joint characteristic function of $p_{\mathrm{VQE},\boldsymbol{\theta}}$, and similarly $\phi_{\mathrm{Haar}}(\mathbf{x} \mid \boldsymbol{\theta})$. Since $\mathbf{U_i}$ are assumed to be i.i.d. ϵ-approximate t-designs under the monomial measure, for any moments $M_{\mathrm{VQE},\boldsymbol{\theta}}, M_{\mathrm{Haar},\boldsymbol{\theta}}$ of degree s of $p_{\mathrm{VQE},\boldsymbol{\theta}}, p_{\mathrm{Haar},\boldsymbol{\theta}}$, respectively, we have that:

$$\left|M_{\mathrm{VQE},\boldsymbol{\theta}} - M_{\mathrm{Haar},\boldsymbol{\theta}}\right| = \mathrm{O}\left(\epsilon \mathbf{1}[s \leq t] + \mathbf{1}[s > t]\right). \tag{50}$$

In particular, for all T sublinear in t,

$$\left|\phi_{\mathrm{VQE}}(\mathbf{x} \mid \boldsymbol{\theta}) - \phi_{\mathrm{Haar}}(\mathbf{x} \mid \boldsymbol{\theta})\right| = \mathrm{O}\left(\epsilon\, \mathrm{poly}(T) + \frac{(3T)^t}{t!}\right) \tag{51}$$

for all $\mathbf{x}$ with $\|\mathbf{x}\|_\infty \leq T$. Similar inequalities hold for the partial derivatives of the joint characteristic functions. Therefore, there exists some $T = \Omega\left(\mathrm{poly}\left(\min\left(t, \frac{1}{\epsilon}\right)\right)\right)$ such that the second bound of Theorem 4 of [43] (with $m = \log(T)$) on the Lévy–Prokhorov distance is $\tilde{\mathrm{O}}\left(\mathrm{poly}\left(\frac{1}{t} + \epsilon\right)\right)$.

Until now, we have considered ansatzes with generic parameter dependence. We now show that up to terms vanishing exponentially quickly in the reverse light cone size l, we can consider a canonical ansatz form WLOG.

Lemma 17 (Canonical form for Hamiltonian agnostic variational loss functions). Let $p_{\mathrm{Haar},\boldsymbol{\theta}}$ be the joint distribution described in Lemma 16. Let $p_{\mathrm{can},\boldsymbol{\theta}}$ be the same for the variational loss function

$$F_{\mathrm{can}}(\boldsymbol{\theta}) = \|\boldsymbol{\alpha}\|_1^{-1} \sum_{i=1}^{A} \alpha_i \langle \mathbf{0}| \mathbf{R}(\boldsymbol{\theta})^\dagger \mathbf{U_i}^\dagger \mathbf{P_i} \mathbf{U_i} \mathbf{R}(\boldsymbol{\theta}) |\mathbf{0}\rangle + 1, \tag{52}$$

where $\mathbf{R}(\boldsymbol{\theta})$ is the product of the parameterized rotations of equation 48. We then have that $p_{\mathrm{Haar},\boldsymbol{\theta}}$ weakly converges to $p_{\mathrm{can},\boldsymbol{\theta}}$, up to an error $\tilde{\mathrm{O}}(\operatorname{poly}\exp(-l))$ in Lévy–Prokhorov distance.

Proof. Let us consider (generally mixed) moments involving random variables of the form:

$$K_{ij}(\boldsymbol{\theta_j}) = \langle \mathbf{0}| \mathbf{U_i}^\dagger \mathbf{W_i}(\boldsymbol{\theta_j})^\dagger \mathbf{P_i} \mathbf{W_i}(\boldsymbol{\theta_j}) \mathbf{U_i} |\mathbf{0}\rangle - \langle \mathbf{0}| \tilde{\mathbf{U}}_{\mathbf{ij}}^\dagger \mathbf{U_i}^\dagger \mathbf{W_i}(\boldsymbol{\theta_j})^\dagger \mathbf{P_i} \mathbf{W_i}(\boldsymbol{\theta_j}) \mathbf{U_i} \tilde{\mathbf{U}}_{\mathbf{ij}} |\mathbf{0}\rangle, \tag{53}$$

where $\mathbf{U_i}, \tilde{\mathbf{U}}_{\mathbf{ij}}$ are i.i.d. Haar random on l qubits. By the asymptotic free independence of Haar random matrices from constant matrices, and the fact that

$$\operatorname{tr}\left(\mathbf{W_i}(\boldsymbol{\theta_j}) \mathbf{P_i} \mathbf{W_i}(\boldsymbol{\theta_j})^\dagger\right) = \operatorname{tr}\left(|\mathbf{0}\rangle\langle \mathbf{0}| - \tilde{\mathbf{U}}_{\mathbf{ij}} |\mathbf{0}\rangle\langle \mathbf{0}| \tilde{\mathbf{U}}_{\mathbf{ij}}^\dagger\right) = 0, \tag{54}$$

we have that any such moment is on the order of $\mathrm{O}(\operatorname{poly}\exp(-l))$ [44]. In particular, it is easy to see that up to an error in Lévy–Prokhorov distance on this order, one can WLOG take $p_{\mathrm{can},\boldsymbol{\theta}}$ as if the gradient and Hessian components had i.i.d. $\mathbf{U_{ij}}$ rather than $\mathbf{U_i}$—for instance, this follows identically to the proof of Lemma 16 with $\epsilon = \mathrm{O}(\operatorname{poly}\exp(-l))$. The result then follows from the unitary invariance of the Haar measure.

We are now able to prove Theorem 15, following essentially the same procedure as proving Theorem 5 of [12].

Proof. By Lemmas 16 and 17, $p_{\mathrm{VQE},\boldsymbol{\theta}}$ weakly converges to $p_{\mathrm{Haar},\boldsymbol{\theta}}$ up to an error $\tilde{\mathrm{O}}\left(\operatorname{poly}\left(\frac{1}{t} + \epsilon + \exp(-l)\right)\right)$ in Lévy–Prokhorov distance. By Corollary 1 of [45], this then proves weak convergence of $p_{\mathrm{VQE},\boldsymbol{\theta}}$ to the corresponding joint distribution of a weighted sum of WHRFs each with 2^{l-1} degrees of freedom, up to an additional error in Lévy–Prokhorov distance exponentially small in l. Weak convergence to $p_{\mathrm{WHRF},\boldsymbol{\theta}}$ then follows from a trivial generalization of Theorem 5 of [12].

Scope of results We now comment on the applicability of the results of [12] on the local minima distribution of WHRFs when Theorem 15 holds. All analysis of the local minima distribution of WHRFs in [12] depends only on the joint distribution $p_{\mathrm{WHRF},\boldsymbol{\theta}}$, up to a change in normalization of the gradient and Hessian by l rather than q that does not contribute to the logarithmic asymptotics (i.e. Theorem 7 of [12]) when $q\log(q) = \mathrm{o}(m)$. Thus, in the discussion of the main text, we take this as an extra assumption. Furthermore, we note that the analysis in the main text holds only up to shifts on the order of $\tilde{\mathrm{O}}\left(\operatorname{poly}\left(\frac{1}{t} + \epsilon + \exp(-l)\right)\right)$ in the joint distribution $p_{\mathrm{WHRF},\boldsymbol{\theta}}$, due to the rate of convergence of Theorem 15. However, shifts on this order do not affect the conclusions of [12] for sufficiently large constant ϵ^{-1}, t. For completeness, we summarize this discussion and known results on the loss landscapes of WHRFs [12] with the following Corollary:

Corollary 18 (Shallow, local VQAs have poor loss landscapes)*.* Let F_{VQE} be a local VQA loss function of the form of equation 47. Assume all coefficients α_i of the Pauli decomposition of $\mathbf{H}$ are $\Theta(1)$, and

$$l\log(n) + q\log(q) = \mathrm{o}\left(2^l A\right). \tag{55}$$

Then $p_{\mathrm{VQE},\boldsymbol{\theta}}$ weakly converges to $p_{\mathrm{WHRF},\boldsymbol{\theta}}$ as in Theorem 15, where the associated WHRF has a fraction superpolynomially small in n of local minima within any constant additive error of the ground state energy.

Proof. The result follows immediately by applying Theorem 7 of [12] to Theorem 15.

Assumptions Let us now discuss in more detail the assumptions made in the course of proving Theorem 15. First, we assume that at least some part of the ansatz circuit scrambles some local region around any measured observable; that is, we assume that the ansatz locally is an ϵ-approximate t design for sufficiently large ϵ^{-1}, t. It is known that shallow, local circuits dimensions exhibit this property, when 2-local Haar random gates are applied [42]; thus, in a practical sense, our results assume that the local gates in any distribution of ansatzes under consideration are approximately Haar random. This is a typical model of Hamiltonian agnostic ansatzes, where the ansatz is chosen independently from the problem Hamiltonian

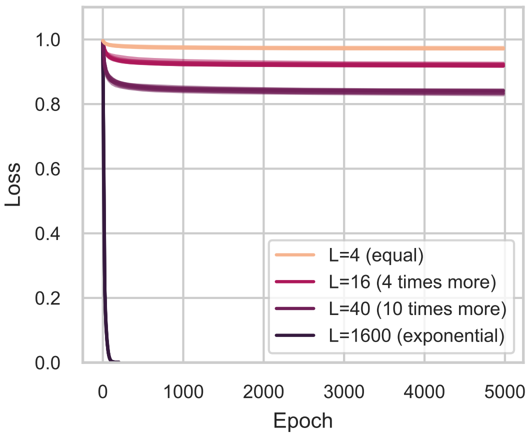

Supplementary Fig. 2: **Teacher-student performance evaluation for the depth L checkerboard ansatz.** Exponential depth is needed to overparamaterize a model to successfully learn a random circuit of the same form. Here, for each student circuit depth denoted by L, 10 randomly initialized 8 qubit student circuits are trained to learn a random $L = 4$ layer teacher circuit drawn from the same ansatz and parameter distribution.

$\mathbf{H}$; see for instance the discussion in the main text and the references therein. The inapplicability of this assumption to Hamiltonian informed ansatzes—particularly for highly symmetric problems—is discussed in more detail in the Discussion of the main text, where we review models that may not suffer from the poor trainability properties we show here.

Our other major assumption is the independence of the $\mathbf{V_i}(\boldsymbol{\theta})$ (up to the repeated use of parameters). Of course, in practice this is almost never true, as otherwise variational optimization would proceed via optimizing each reverse light cone independently. However, given a problem Hamiltonian $\mathbf{H}$ and a shallow ansatz, one can consider a subset of Pauli operators in the Pauli decomposition of $\mathbf{H}$ such that their reverse light cones do not overlap. There is little reason to believe that the loss landscape of this simplified problem should be any more difficult to optimize over than the full problem. We therefore suspect that this assumption is little more than a technical requirement. A similar generalization one could consider is taking the parameters of each $\mathbf{V_i}$ to being almost entirely independent of one another (though not entirely independent, as one could then optimize each subproblem independently, and n would no longer an accurate measure of the size of the problem). However, in this regime we expect the "effective" overparameterization ratio γ to go as $\left(\frac{l}{2^l}\right)^A$, as the problem essentially reduces to simultaneously optimizing A loss functions. For $A \sim n$, for instance, this decays exponentially in n, and thus we believe that models of this form are also not trainable.

Supplementary Note 5– Additional Numerical Experiments

Supplementary Note 5.1– Teacher Student Learning With Checkerboard Ansatzes

One particular challenge with quantum variational learning is that an overparameterized model needs more parameters than the dimension of the quantum input state (exponential in the number of qubits), whereas classically, overparameterization with respect to the size of the data set typically suffices [36, 46, 47]. To illustrate this phenomenon, we consider learning states generated by random shallow checkerboard circuits (denoted the teacher circuit) using checkerboard circuits of the same or more depth (denoted the student circuit). The data set used to train the circuit consist of 512 pairs of inputs randomly drawn from computational basis states with their corresponding output state taken from applying the input state to the teacher

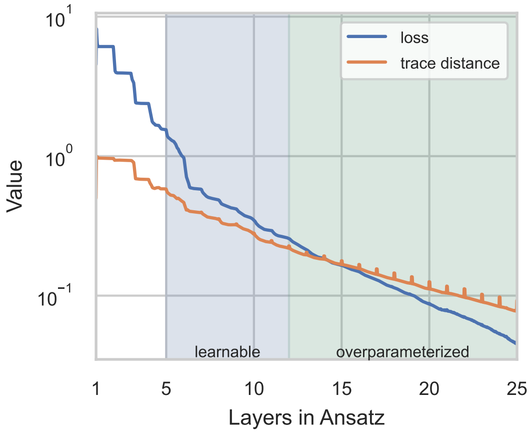

Supplementary Fig. 3: **Layer-wise learning evaluation.** When optimizing in a layer-wise fashion, the VQE algorithm converges to a local minimum at each layer until the overparameterized regime where the loss function steadily decreases regardless of the number of layers. Even in the learnable regime where the checkerboard ansatz is capable of expressing the global minima, the ansatz is still unable to find the correct parameters for this global minimum. Bumps in the loss function appear due to small instabilities in training immediately after adding a layer.

circuit. We use the loss $\ell(|\psi\rangle, |\phi\rangle) = 1 - |\langle\psi|\phi\rangle|^2$ to measure the success of learning. Note that, though this is a global loss metric, gradients are analytically calculated to precision sufficient enough to obtain accurate values of the gradients for the relatively small number of qubits considered here.

As shown in Supplementary Fig. 2, exponential depth (and number of parameters) is needed to always successfully learn the data generated by a shallow checkerboard circuit of 4 layers. We considered ansatzes only over 8 qubits, which is small enough to be able to feasibly overparameterize the models in our simulations. For fewer qubits and shallower circuits, we found that learning with equal numbers of qubits and layers was sometimes successful; but unsurprisingly, as we show in the main text, learning becomes much harder as qubits are added.

Supplementary Note 5.2– Random VQE Model

Here, we empirically analyze the performance of a layer-wise optimizer trained on the random VQE task in the main text. In Supplementary Fig. 3, we train an 11 qubit ansatz using a layer-wise optimizer [48, 49], which initially trains a single layer of the ansatz and adds layers after every 5000 steps to continually add expressiveness. The target Hamiltonian $\mathbf{H}_t$ here has 4 layers of perturbations applied to it. Although layer-wise optimizers can avoid issues with barren plateaus [48], our numerical findings clearly show that this does not guarantee the algorithm will avoid traps in the landscape. After 5 layers, the ansatz has enough parameters to capably express the global optimum (denoted by the label "learnable"), but nevertheless stalls in optimizing to the ground state. Not until there are at least 12 layers, enough to overparameterize the ansatz with respect to the Hilbert space dimension, does learning smoothly converge to the globally optimal solution.

Supplementary Note 5.3– XYZ Hamiltonian Model

All of the numerical experiments performed elsewhere studied settings where the optimization was performed to numerical precision, two qubit gates were fully parameterized, and the existence of a global minimum at zero loss was guaranteed. The analysis there focused on answering the question of whether convergence to the

global minimum is empirically likely to be observed. To study a more realistic setting where such favorable conditions cannot be guaranteed but there still exists hope of some good convergence properties, we turn now to the problem of trying to variationally obtain the ground state of an approximately translationally invariant Heisenberg XYZ Hamiltonian [50]. Similar Hamiltonians have been studied and analyzed in previous works related to VQE [51, 52]. We perform experiments both with and without Gaussian noise added to the gradients to account for shot noise on a quantum computer.

The particular target Hamiltonian we aim to optimize is one where qubits are placed on a 2-dimensional grid and interaction terms take place between neighboring qubits. The Hamiltonian takes the form

$$\mathbf{H} = \sum_i \mathbf{Z}_i + \sum_{\langle i,j \rangle} \alpha_{ij} \mathbf{Z}_i \otimes \mathbf{Z}_j + \sum_{\langle i,j \rangle} \beta_{ij} \left(\mathbf{X}_i \otimes \mathbf{X}_j + 0.66 \mathbf{Y}_i \otimes \mathbf{Y}_j \right), \tag{56}$$

where $\langle i, j \rangle$ sums over the neighboring qubits i and j in the grid and α_{ij} and β_{ij} are random numbers drawn from the normal distribution with standard deviations set to 0.25 and means set to 1 and 3, respectively.

Supplementary Table 1: Error in energy from the ground state (normalized by the magnitude of the ground state energy), and trace distance from the ground state, of a VQE optimizing the Heisenberg XYZ model. Results are averaged across 12 random initializations of the experiment for each entry in the table. Note the poor performance of VQE, particularly at the larger problem sizes.

		energy error			trace distance		
	grid size	3×2	5×2	7×2	3×2	5×2	7×2
layers	shots						
3	10000	0.459	0.512	0.504	0.983	0.999	1.000
	400	0.456	0.501	0.392	0.983	0.999	1.000
	inf	0.466	0.512	0.395	0.981	0.999	1.000
9	10000	0.269	0.358	0.351	0.750	0.965	0.998
	400	0.343	0.434	0.386	0.845	0.991	0.994
	inf	0.245	0.350	0.344	0.659	0.924	0.993
15	10000	0.104	0.293	0.303	0.428	0.894	0.997
	400	0.180	0.356	0.318	0.577	0.965	0.987
	inf	0.054	0.244	0.251	0.293	0.842	0.968
21	10000	0.008	0.201	0.214	0.162	0.799	0.982
	400	0.043	0.277	0.247	0.269	0.882	0.984
	inf	0.011	0.178	0.162	0.151	0.747	0.933
27	10000	0.009	0.177	0.200	0.152	0.752	0.976
	400	0.034	0.254	0.251	0.244	0.882	0.976
	inf	0.010	0.122	0.129	0.136	0.663	0.948

As shown in Table 1, finding the ground state of the XYZ hamiltonian is in general challenging using the ansatz considered. For few layers, the ansatz is not expressible enough to find the target and converges to a poor critical point. For many layers, the VQE algorithm tends to converge to a better optimum, but issues with barren plateaus can begin to arise as indicated by the comparison in performance with assuming infinite shots vs. finite shots.

Supplementary Note 6– Details of Numerical Experiments

All experiments were performed in Python using the PyTorch [53] package to perform automatic differentiation. Computation was performed on Nvidia RTX™ A6000 GPUs. Important hyperparameters for the experiments are listed in Table 2. Unless otherwise stated, all gradients were calculated using analytic formulas for automatic differentiation with computer precision (32 bit floating point). Therefore, issues with

Supplementary Table 2: List of parameter counts, optimizers, and learning rates for the various ansatzes and experiments. L denotes the number of layers and n the number of qubits.

Ansatz	Experiment	# Parameters	Optimizer	Learning Rate
QCNN	Teacher-Student	$16\lceil\log_2 n\rceil = \mathrm{O}(\log n)$	Adam	0.001
Checkerboard	Teacher-Student	$32L\lfloor\frac{n}{2}\rfloor = \mathrm{O}(nL)$	Adam	0.001 (underparameterized)
				0.0001 (overparameterized)
	Random VQE (GD)	$128\lfloor\frac{n}{2}\rfloor = \mathrm{O}(n)$	vanilla GD	0.01
	Random VQE (Adam)	$128\lfloor\frac{n}{2}\rfloor = \mathrm{O}(n)$	Adam	0.003
	Adaptive VQE	$160L = \mathrm{O}(L)$	Adam	0.002 (5% reduction each layer)
XYZ ansatz	XYZ Hamiltonian VQE	$7\lfloor\frac{L}{3}\rfloor = \mathrm{O}(L)$	Adam	0.007 (halved every 1000 steps)

decaying gradients and barren plateaus do not appear in these simulations for the relatively small number of qubits considered. Gradient based optimization was performed using vanilla gradient descent or the Adam optimizer [54], a popular and effective algorithm for training deep neural networks. We tested other optimizers as well and found no noticeable difference in performance.

Loss surface plot To generate this plot, we chart the loss landscape at initialization of training in the teacher-student setup of the main text for the 14 qubit QCNN circuit. The teacher and student circuit were both initialized as described in Supplementary Note 6.1.

The loss is plotted along two normalized directions of the parameter landscape. Normalization is applied individually to the 3 filters of the 14 qubit QCNN. We loosely follow the "filter-wise" normalization strategy of [55], where we first generate a random direction by drawing a value for each parameter from an i.i.d. standard normal distribution. Then, we divide values for the parameters in a given layer by the Frobenius norm of the matrix for the corresponding layer.

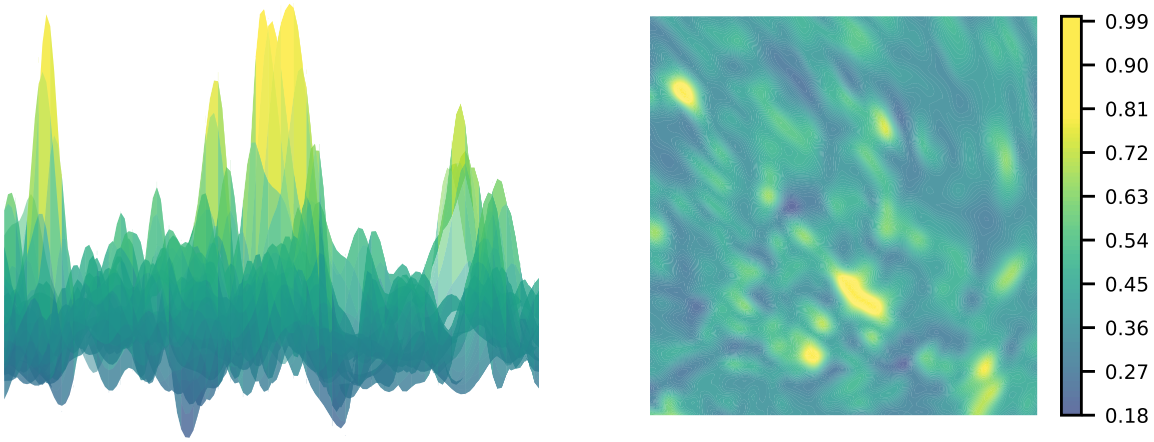

Supplementary Fig. 4: **Randomly initialized QCNN evaluation.** Loss landscape of the QCNN experiment replicated from the main text, except the initialization of the student circuit is randomly chosen. Here, the global minimum is likely far away and the landscape also appears "bumpy"; all local minima in the region considered here are far from the global optimum.

In the loss surface plot of the main text, we plot the mean squared error loss for the teacher-student task for a batch size of 128 randomly chosen computational basis states. The legend in the plot is shown relative to the maximum value of the loss in the range considered. A value of 0 here corresponds to the loss at the global minimum. The middle of the plot corresponds to the exact parameters of the teacher circuit, and hence, is a global minimum. This setting is, in a sense, an optimistic setting since initialization is near a global minimum. For comparison, we include in Supplementary Fig. 4 an example of a loss surface where the student circuit is not initialized near the parameters of the teacher circuit. As is evident in this setting, no longer is there a global minimum in the parameter region considered, and the landscape also appears to be filled with traps.

Supplementary Note 6.1– QCNN Experiments

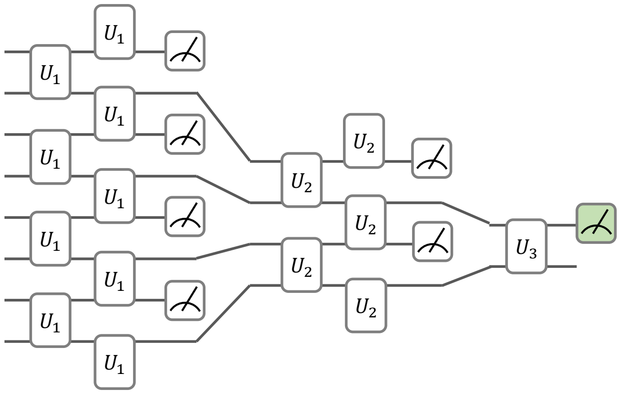

Supplementary Fig. 5: **QCNN ansatz for 8 qubits.** Layers of shared 2-local unitary transformations are applied followed by measurement of every other qubit. Gates at the edge of the circuit above are applied in a cyclic fashion (i.e. the top and bottom qubit interact). The measurement colored in green is the measurement outcome whose probability we aim to predict in the teacher-student setup. Generically for n qubits, this ansatz has depth $\lceil \log_2 n \rceil$. During training, the 2-local unitaries are fully parameterized for our simulations.

The quantum convolutional neural network (QCNN) is an ansatz originally proposed in [56]. This ansatz features parameter sharing across gates in a single layer. The form of this circuit is provided in Supplementary Fig. 5. In our experiments, we use the same form of the 2-local ansatz as in [56] and also studied in [57]. Between convolutional layers, we include no controlled unitary operations based on the measurement outcomes. In learning settings, we fully parameterize the 2-local unitaries in the skew Hermitian basis of the unitary Lie algebra. To achieve this, we train directly over parameter entries of a matrix $\mathbf{M}$ and apply $e^{\mathbf{H}}$, where $\mathbf{H} = \mathbf{M} - \mathbf{M}^\dagger$, to perform the resulting unitary transformation. Entries of the matrix $\mathbf{M}$ were initialized i.i.d. from a standard normal distribution.

For the teacher-student experiments in the main text, we aim to predict the outcome of the final green measurement depicted in Supplementary Fig. 5 for 512 randomly chosen computational basis states. For n qubits, the QCNN ansatz for both the teacher and student circuits have $16\lceil \log_2 n \rceil$ parameters which is a relatively small number compared to the dimension of the Hilbert space. All networks were trained for 5000 epochs and a learning rate of 0.001 using the Adam optimizer.

Supplementary Note 6.2– Checkerboard Ansatz

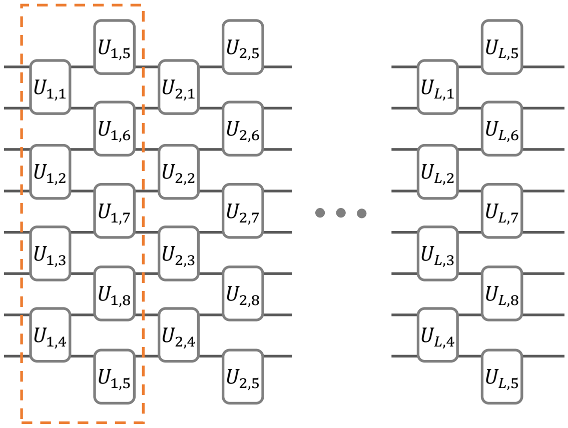

Supplementary Fig. 6: **Checkerboard ansatz for 8 qubits and L layers.** Gates at the edge of the circuit above are applied in a cyclic fashion (i.e. the top and bottom qubit interact). Generically for n qubits, this ansatz has $32L\lfloor n/2 \rfloor$ parameters. During training, the 2-local unitaries are fully parameterized for our simulations.

The checkerboard circuit applies gates in a one-dimensional lattice as shown in Supplementary Fig. 6. As in the QCNN experiments, we train directly over parameter entries of a matrix $\mathbf{M}$ and apply $e^{\mathbf{H}}$, where $\mathbf{H} = \mathbf{M} - \mathbf{M}^\dagger$, to perform the resulting unitary transformation. Since the exponential map from the Lie algebra is surjective onto the unitary group, this parameterization is capable of expressing any unitary matrix. Entries of the matrix $\mathbf{M}$ were initialized i.i.d. from a standard normal distribution.

For the teacher-student simulations of the main text, we train networks over 512 randomly chosen computational basis states which is more than the dimension of the Hilbert space and enough information to recover the full unitary transformation. Optimization was performed using the Adam optimizer and a batch size of 128. Networks were trained for 5000 epochs and training was stopped if the loss fell below 0.001 which only occured for the overparameterized setting. We observed that for fewer than 8 qubits, training was successful with very small probability in the underparameterized setting.

Supplementary Note 6.3– VQE Experiments on Random Hamiltonians

For all of our VQE experiments, the target Hamiltonian $\mathbf{H}_t$ was constructed by conjugating a local Hamiltonian of n qubits equal to $\sum_{i=1}^n \mathbf{Z}_i$ with alternating layers of products of two-qubit unitaries $\mathbf{U}_1$ and $\mathbf{U}_2$. That is, $\mathbf{H}_t$ takes the form below as copied from the main text:

$$\mathbf{H}_t = \left(\mathbf{U}_2^\dagger \mathbf{U}_1^\dagger\right)^L \left[\sum_{i=1}^n \mathbf{Z}_i\right] (\mathbf{U}_1 \mathbf{U}_2)^L + n\mathbf{I}. \tag{57}$$

$\mathbf{U}_1$ and $\mathbf{U}_2$ are the tensor product of two-qubit unitaries which for n even take the form:

$$\begin{aligned} \mathbf{U}_1 &= \mathbf{U}_1^{(1,2)} \otimes \mathbf{U}_1^{(3,4)} \otimes \cdots \otimes \mathbf{U}_1^{(n-1,n)} \\ \mathbf{U}_2 &= \mathbf{U}_2^{(2,3)} \otimes \mathbf{U}_2^{(4,5)} \otimes \cdots \otimes \mathbf{U}_2^{(n,n+1)}, \end{aligned} \tag{58}$$

where superscripts above indicate the pair of qubits each 2-local unitary acts on and indexing is taken mod n. Each 2 local unitary is drawn from the distribution $e^{\mathbf{H}}$, where $\mathbf{H} = \mathbf{G} - \mathbf{G}^\dagger$, and each $\mathbf{G}$ is a 4×4 matrix with entries drawn i.i.d. from a random normal distribution. Trained unitaries in the checkerboard ansatz are also initialized in this fashion. Optimization is then performed directly on the entries of the matrix in the Lie algebra which form a complete basis for all of the 2-local unitaries.

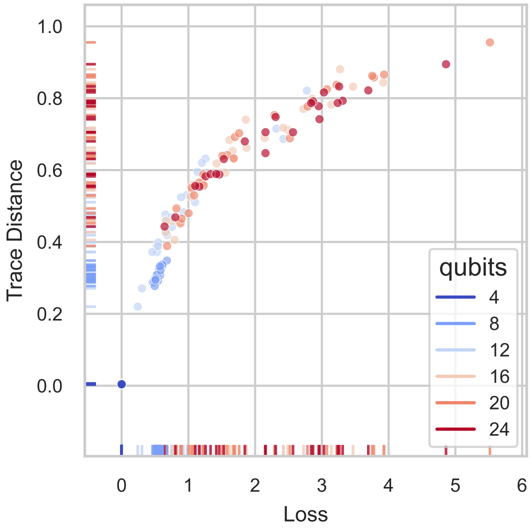

Supplementary Fig. 7: Adam performance evaluation. Scatter plot showing the values of the loss and trace distance of the final VQE state after 30000 steps of optimization using the Adam optimizer shows that the algorithm converges to poorer local minima as the number of qubits grows. Setting is replicated from the VQE loss experiment from the main text, with the sole change of the optimizer from gradient descent to Adam.

In the VQE loss experiment of the main text, each VQE instance was optimized for 30000 steps using a vanilla gradient descent optimizer with a learning rate of 0.01. For completeness, we replicate this plot with the Adam optimizer in Supplementary Fig. 7 and unsurprisingly observe similar convergence results. All calculations were performed to computer precision, which provides a best-case setting for optimization via real quantum hardware, since gradients and loss function values would have to be calculated using less precise sampling methods on actual quantum computers. In layer-wise VQE experiment of the main text, optimization is performed using an adaptive VQE algorithm similar to the one in [49]. Here, a checkerboard ansatz is initialized as a single layer and optimization is performed layer-wise. We set $n = 11$ and small enough such that it is computationally feasible to overparameterize the ansatz. Each 5000 steps of optimization, a layer is added to the ansatz and initialized to the identity mapping. Each additional layer adds 160 trainable parameters to the ansatz. After each layer is added, the learning rate is multiplied by 0.95 to make the training more stable with more parameters. At each point in time, all parameters of the ansatz across all layers are trained. For aesthetic purposes and to see the course of training without significant jumps in the plot, we plot a moving average of the values across 10 sequential datapoints in the main text.

Supplementary Note 6.4– VQE experiments on XYZ Hamiltonian

For the XYZ Hamiltonian model empirically analyzed in Supplementary Note 5.3, we implemented a gate-based ansatz which is fully parameterized in the single qubit gates and parameterized only with Pauli $\mathbf{Z} \otimes \mathbf{Z}$ terms for two qubit gates. The form of the ansatz is depicted in Supplementary Fig. 8. Since the Hamiltonian of the model is approximately translationally invariant in both directions, we implemented

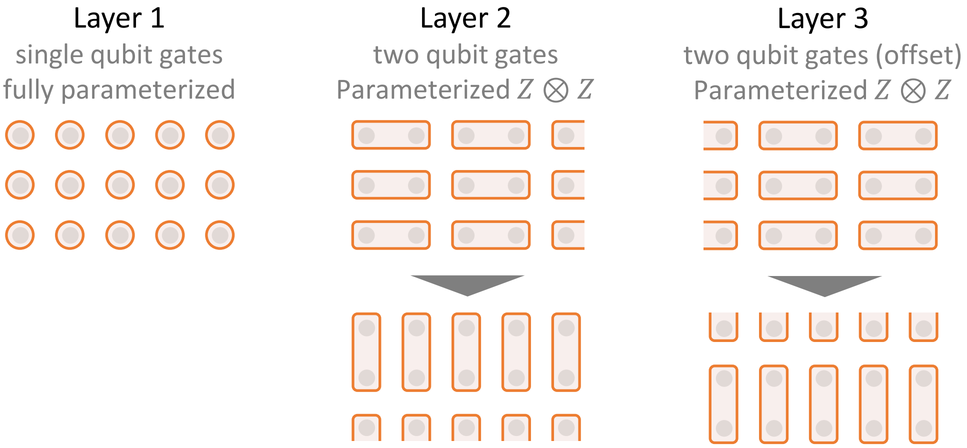

Supplementary Fig. 8: **Form of the ansatz used for the XYZ Hamiltonian VQE experiments in Supplementary Note 5.3.** Here, alternating blocks of three layers are composed onto each other. The first layer in each block is a fully parameterized single qubit gate. The next two layers are parameterized Pauli $\mathbf{Z} \otimes \mathbf{Z}$ terms to connect all neighboring qubits. Parameters are shared across a layer to better address the near translationally invariance in the model.

sharing of parameters across a layer. Parameters were initialized as random normal variables. Each instance was optimized using the Adam optimizer [54] for 5000 steps. The learning rate was initially set to 0.007 and halved every 1000 steps. For calculations of the trace distance to the ground state, the ground state of the Hamiltonian was calculated by performing an eigendecomposition of the complete Hamiltonian. To account for shot noise, random centered Gaussian noise with standard deviation equal to $1/\sqrt{\# \text{ shots}}$ was added to gradients with respect to the parameters.

Supplementary Note 7– Untrainability Beyond Gradient Descent

One may wish to avoid local minima by changing the loss function or performing more advanced versions of gradient-based optimizers. Here, we gives heuristic reasons why these two adjustments will likely not fix any issues of untrainability.

First, we examine changes in the loss function. This is commonly done to avoid barren plateaus and make gradients easier to compute. Let us assume that $\mathcal{L}(\boldsymbol{\theta})$ is our original loss function (as a function of the parameters $\boldsymbol{\theta}$), which is changed to a new loss function $\tilde{\mathcal{L}}(\boldsymbol{\theta})$. Typically, $\tilde{\mathcal{L}}(\boldsymbol{\theta})$ is chosen so that it upper and lower bounds $\mathcal{L}(\boldsymbol{\theta})$, i.e. $C\tilde{\mathcal{L}}(\boldsymbol{\theta}) \leq \mathcal{L}(\boldsymbol{\theta}) \leq D\tilde{\mathcal{L}}(\boldsymbol{\theta})$ for some constants C, D. This guarantees convergence in both metrics when changing the loss function and is the case for e.g. local versions of the inner product and the quantum earth mover's (EM) distance [58, 59]. Now, let us assume that every continuous path from a local minimum at $\boldsymbol{\theta_l}$ to the global minimum $\boldsymbol{\theta}^*$ must increase the loss function by a factor $M > D/C$, i.e. there exists a point in the path that has value at least $M\mathcal{L}(\boldsymbol{\theta_l})$. Then, in the new loss function $\tilde{\mathcal{L}}(\boldsymbol{\theta_l}) \leq \mathcal{L}(\boldsymbol{\theta_l})/C$. Furthermore, at some point in any continuous path, $\mathcal{L}(\boldsymbol{\theta}) > M\mathcal{L}(\boldsymbol{\theta_l})$ which implies that at that point $\tilde{\mathcal{L}}(\boldsymbol{\theta}) \geq \mathcal{L}(\boldsymbol{\theta})/D > M\mathcal{L}(\boldsymbol{\theta_l})/D = \mathcal{L}(\boldsymbol{\theta_l})/C$. Thus, $\boldsymbol{\theta_l}$ is not within a convex region around the global optimum. This may be too restrictive of an assumption since local minima can often be very shallow, but it also seems to be backed by experiments.

Second, we consider changing the optimization algorithm to a second order optimization algorithm such as in [60]. These algorithms perform gradient descent by applying a transformation to the gradient of the form:

$$\boldsymbol{\theta_{t+1}} = \boldsymbol{\theta_t} - \mu \boldsymbol{\Sigma}^+ \nabla_{\boldsymbol{\theta_t}} \mathcal{L}(\boldsymbol{\theta_t}), \tag{59}$$

where $\mathbf{\Sigma}$ incorporates our second order term, e.g. the Hessian or Fubini–Study metric tensor, and $\mathbf{\Sigma}^+$ is its pseudoinverse. Clearly, in the above, this does not allow one to escape a local minima. Setting $\boldsymbol{\theta_t} = \boldsymbol{\theta}^*$ above sets the gradient term to zero, and one again one is stuck in a local minimum.

Though other training methods exist, it is not clear *a priori* why they should succeed. For example, training in a layer-wise fashion also does not work as [59, 48, 61] show. Finally, note that the above methods can be very effective at alleviating barren plateaus. In fact, changes in metric and second order optimization methods are often precisely designed to fix this issue. Nevertheless, these methods only provably converge to the global optimum in convex or close to convex settings, which is not the case for essentially all variational quantum models.

Supplementary References

[1] A. Peruzzo, J. McClean, P. Shadbolt, M.-H. Yung, X.-Q. Zhou, P. J. Love, A. Aspuru-Guzik, and J. L. O'brien, Nat. Commun. **5**, 4213 (2014).

[2] L. Bottou and O. Bousquet, in *Proceedings of the 20th International Conference on Neural Information Processing Systems*, NIPS'07 (Curran Associates Inc., Red Hook, NY, USA, 2007) p. 161–168.

[3] K. Nakaji and N. Yamamoto, Quantum **5**, 434 (2021).

[4] S. Sim, P. D. Johnson, and A. Aspuru-Guzik, Adv. Quantum Technol. **2**, 1900070 (2019).

[5] Y. Du, M.-H. Hsieh, T. Liu, and D. Tao, Phys. Rev. Research **2**, 033125 (2020).

[6] H. Shen, P. Zhang, Y.-Z. You, and H. Zhai, Phys. Rev. Lett. **124**, 200504 (2020).

[7] M. C. Caro, H.-Y. Huang, M. Cerezo, K. Sharma, A. Sornborger, L. Cincio, and P. J. Coles, Nat. Commun. **13**, 4919 (2022).

[8] Y. Du, Z. Tu, X. Yuan, and D. Tao, Phys. Rev. Lett. **128**, 080506 (2022).

[9] C. Zhang, S. Bengio, M. Hardt, B. Recht, and O. Vinyals, Commun. ACM **64**, 107 (2021).

[10] B. Neyshabur, S. Bhojanapalli, D. Mcallester, and N. Srebro, in *Advances in Neural Information Processing Systems*, Vol. 30, edited by I. Guyon, U. V. Luxburg, S. Bengio, H. Wallach, R. Fergus, S. Vishwanathan, and R. Garnett (Curran Associates, Inc., 2017).

[11] H. Maennel, I. Alabdulmohsin, I. Tolstikhin, R. J. N. Baldock, O. Bousquet, S. Gelly, and D. Keysers, in *Proceedings of the 34th International Conference on Neural Information Processing Systems*, NIPS'20 (Curran Associates Inc., Red Hook, NY, USA, 2020).

[12] E. R. Anschuetz, in *International Conference on Learning Representations* (2022).

[13] R. Wiersema, C. Zhou, Y. de Sereville, J. F. Carrasquilla, Y. B. Kim, and H. Yuen, PRX Quantum **1**, 020319 (2020).

[14] B. T. Kiani, S. Lloyd, and R. Maity, Learning unitaries by gradient descent (2020), arXiv:2001.11897 [quant-ph] .

[15] B. Applebaum, B. Barak, and D. Xiao, in *2008 49th Annual IEEE Symposium on Foundations of Computer Science* (2008) pp. 211–220.

[16] M. Kearns, J. ACM **45**, 983 (1998).

[17] S. Arunachalam, A. B. Grilo, and H. Yuen, Quantum statistical query learning (2020), arXiv:2002.08240 [quant-ph] .

[18] B. Szörényi, in *Algorithmic Learning Theory*, edited by R. Gavaldà, G. Lugosi, T. Zeugmann, and S. Zilles (Springer Berlin Heidelberg, Berlin, Heidelberg, 2009) pp. 186–200.

[19] L. G. Valiant, Commun. ACM **27**, 1134–1142 (1984).

[20] L. Reyzin, Statistical queries and statistical algorithms: Foundations and applications (2020), arXiv:2004.00557 [cs.LG] .

[21] M. Hinsche, M. Ioannou, A. Nietner, J. Haferkamp, Y. Quek, D. Hangleiter, J.-P. Seifert, J. Eisert, and R. Sweke, Learnability of the output distributions of local quantum circuits (2021), arXiv:2110.05517 [quant-ph] .

[22] A. Gollakota and D. Liang, Quantum **6**, 640 (2022).

[23] I. Diakonikolas, D. M. Kane, V. Kontonis, and N. Zarifis, in *Proceedings of Thirty Third Conference on Learning Theory*, Proceedings of Machine Learning Research, Vol. 125, edited by J. Abernethy and S. Agarwal (PMLR, 2020) pp. 1514–1539.

[24] S. Goel, A. Gollakota, Z. Jin, S. Karmalkar, and A. Klivans, in *Proceedings of the 37th International Conference on Machine Learning*, Proceedings of Machine Learning Research, Vol. 119, edited by H. D. III and A. Singh (PMLR, 2020) pp. 3587–3596.

[25] S. Goel, A. Gollakota, and A. Klivans, in *Proceedings of the 34th International Conference on Neural Information Processing Systems*, NIPS'20 (Curran Associates Inc., Red Hook, NY, USA, 2020).

[26] S. Chen, A. Gollakota, A. R. Klivans, and R. Meka, Hardness of noise-free learning for two-hidden-layer neural networks (2022), arXiv:2202.05258 [cs.LG] .

[27] I. Diakonikolas, D. Kane, and N. Zarifis, in *Advances in Neural Information Processing Systems*, Vol. 33, edited by H. Larochelle, M. Ranzato, R. Hadsell, M. Balcan, and H. Lin (Curran Associates, Inc., 2020) pp. 13586–13596.

[28] Y. LeCun, Y. Bengio, and G. Hinton, Nature **521**, 436 (2015).

[29] S. Chen, A. R. Klivans, and R. Meka, in *2021 IEEE 62nd Annual Symposium on Foundations of Computer Science (FOCS)* (IEEE Computer Society, Los Alamitos, CA, USA, 2022) pp. 696–707.

[30] A. Andoni, R. Panigrahy, G. Valiant, and L. Zhang, in *Proceedings of the Twenty-Fifth Annual ACM-SIAM Symposium on Discrete Algorithms*, SODA '14 (Society for Industrial and Applied Mathematics, USA, 2014) pp. 500–510.

[31] J. Napp, Quantifying the barren plateau phenomenon for a model of unstructured variational ansätze (2022), arXiv:2203.06174 [quant-ph] .

[32] B. Collins, Int. Math. Res. Not. **2003**, 953 (2003).

[33] B. Collins and P. Śniady, Commun. Math. Phys. **264**, 773 (2006).

[34] A. Blum, M. Furst, J. Jackson, M. Kearns, Y. Mansour, and S. Rudich, in *Proceedings of the Twenty-Sixth Annual ACM Symposium on Theory of Computing*, STOC '94 (Association for Computing Machinery, New York, NY, USA, 1994) pp. 253–262.

[35] H. Buhrman, R. Cleve, J. Watrous, and R. de Wolf, Phys. Rev. Lett. **87**, 167902 (2001).

[36] A. Choromanska, M. Henaff, M. Mathieu, G. B. Arous, and Y. LeCun, in *Proceedings of the Eighteenth International Conference on Artificial Intelligence and Statistics*, Proceedings of Machine Learning Research, Vol. 38, edited by G. Lebanon and S. V. N. Vishwanathan (PMLR, San Diego, California, USA, 2015) pp. 192–204.

[37] P. Chaudhari and S. Soatto, On the energy landscape of deep networks (2017), arXiv:1511.06485 [cs.LG] .

[38] J. R. McClean, S. Boixo, V. N. Smelyanskiy, R. Babbush, and H. Neven, Nat. Commun. **9**, 4812 (2018).

[39] M. Cerezo, A. Sone, T. Volkoff, L. Cincio, and P. J. Coles, Nat. Commun. **12**, 1791 (2021).

[40] M. Schuld, V. Bergholm, C. Gogolin, J. Izaac, and N. Killoran, Phys. Rev. A **99**, 032331 (2019).

[41] A. Mari, T. R. Bromley, and N. Killoran, Phys. Rev. A **103**, 012405 (2021).

[42] A. Harrow and S. Mehraban, Approximate unitary t-designs by short random quantum circuits using nearest-neighbor and long-range gates (2018), arXiv:1809.06957 [quant-ph] .

[43] A. Y. Zaitsev, J. Sov. Math. **27**, 3070 (1984).

[44] D. Voiculescu, Invent. Math. **104**, 201 (1991).

[45] T. Jiang, Probab. Theory Relat. Fields **131**, 121 (2005).

[46] Y. Li and Y. Liang, in *Proceedings of the 32nd International Conference on Neural Information Processing Systems*, NIPS'18 (Curran Associates Inc., Red Hook, NY, USA, 2018) pp. 8168–8177.

[47] S. Arora, S. S. Du, W. Hu, Z. Li, R. Salakhutdinov, and R. Wang, in *Proceedings of the 33rd International Conference on Neural Information Processing Systems* (Curran Associates Inc., Red Hook, NY, USA, 2019) pp. 8141–8150.

[48] A. Skolik, J. R. McClean, M. Mohseni, P. van der Smagt, and M. Leib, Quantum Mach. Intell. **3**, 5 (2021).

[49] H. R. Grimsley, S. E. Economou, E. Barnes, and N. J. Mayhall, Nat. Commun. **10**, 3007 (2019).

[50] W. Heisenberg, Z. Phys. **49**, 619 (1928).

[51] D. Wecker, M. B. Hastings, and M. Troyer, Phys. Rev. A **92**, 042303 (2015).

[52] C. Cade, L. Mineh, A. Montanaro, and S. Stanisic, Phys. Rev. B **102**, 235122 (2020).

[53] A. Paszke, S. Gross, F. Massa, A. Lerer, J. Bradbury, G. Chanan, T. Killeen, Z. Lin, N. Gimelshein, L. Antiga, A. Desmaison, A. Köpf, E. Yang, Z. DeVito, M. Raison, A. Tejani, S. Chilamkurthy, B. Steiner, L. Fang, J. Bai, and S. Chintala, in *Proceedings of the 33rd International Conference on Neural Information Processing Systems* (Curran Associates Inc., Red Hook, NY, USA, 2019).

[54] D. P. Kingma and J. Ba, Adam: A method for stochastic optimization (2014), arXiv:1412.6980 [cs.LG] .

[55] H. Li, Z. Xu, G. Taylor, C. Studer, and T. Goldstein, in *Advances in Neural Information Processing Systems*, Vol. 31, edited by S. Bengio, H. Wallach, H. Larochelle, K. Grauman, N. Cesa-Bianchi, and R. Garnett (Curran Associates, Inc., 2018).

[56] I. Cong, S. Choi, and M. D. Lukin, Nat. Phys. **15**, 1273 (2019).

[57] A. Pesah, M. Cerezo, S. Wang, T. Volkoff, A. T. Sornborger, and P. J. Coles, Phys. Rev. X **11**, 041011 (2021).

[58] G. De Palma, M. Marvian, D. Trevisan, and S. Lloyd, IEEE Trans. Inf. Theory **67**, 6627 (2021).

[59] B. T. Kiani, G. De Palma, M. Marvian, Z.-W. Liu, and S. Lloyd, Quantum earth mover's distance: A new approach to learning quantum data (2021), arXiv:2101.03037 [quant-ph] .

[60] J. Stokes, J. Izaac, N. Killoran, and G. Carleo, Quantum **4**, 269 (2020).

[61] E. Campos, A. Nasrallah, and J. Biamonte, Phys. Rev. A **103**, 032607 (2021).
